# Classification of early-MCI patients from healthy controls using evolutionary optimization of graph measures of resting-state fMRI, for the Alzheimer’s disease neuroimaging initiative

**DOI:** 10.1371/journal.pone.0267608

**Published:** 2022-06-21

**Authors:** Jafar Zamani, Ali Sadr, Amir-Homayoun Javadi

**Affiliations:** 1 School of Electrical Engineering, Iran University of Science and Technology, Tehran, Iran; 2 School of Psychology, University of Kent, Canterbury, United Kingdom; 3 School of Rehabilitation, Tehran University of Medical Sciences, Tehran, Iran; Nathan S Kline Institute, UNITED STATES

## Abstract

Identifying individuals with early mild cognitive impairment (EMCI) can be an effective strategy for early diagnosis and delay the progression of Alzheimer’s disease (AD). Many approaches have been devised to discriminate those with EMCI from healthy control (HC) individuals. Selection of the most effective parameters has been one of the challenging aspects of these approaches. In this study we suggest an optimization method based on five evolutionary algorithms that can be used in optimization of neuroimaging data with a large number of parameters. Resting-state functional magnetic resonance imaging (rs-fMRI) measures, which measure functional connectivity, have been shown to be useful in prediction of cognitive decline. Analysis of functional connectivity data using graph measures is a common practice that results in a great number of parameters. Using graph measures we calculated 1155 parameters from the functional connectivity data of HC (n = 72) and EMCI (n = 68) extracted from the publicly available database of the Alzheimer’s disease neuroimaging initiative database (ADNI). These parameters were fed into the evolutionary algorithms to select a subset of parameters for classification of the data into two categories of EMCI and HC using a two-layer artificial neural network. All algorithms achieved classification accuracy of 94.55%, which is extremely high considering single-modality input and low number of data participants. These results highlight potential application of rs-fMRI and efficiency of such optimization methods in classification of images into HC and EMCI. This is of particular importance considering that MRI images of EMCI individuals cannot be easily identified by experts.

## Introduction

Alzheimer’s disease (AD) is the most common type of dementia, with around 50 million patients worldwide [1,2]. AD is usually preceded by a period of mild cognitive impairment (MCI) [3,4]. Identifying the subjects with MCI could be an effective strategy for early diagnosis and delay the progression of AD towards irreversible brain damage [5–7]. While researchers were fairly successful in diagnosis of AD, researchers were significantly less successful in diagnosis of MCI [8–11]. In particular, detection of early stages of MCI (EMCI) has been proven to be very challenging [12–14]. Therefore, in this study we propose a novel method based on evolutionary algorithms to select a subset of graph features calculated from functional connectivity data to discriminate between healthy participants (HC) and EMCI.

It has been shown that the brain goes through many functionally, structurally and physiologically changes prior to any obvious behavioral symptoms in AD [15–17]. Therefore, many approaches have been devised based on biomarkers to distinguish between HC, and different stages of MCI, and AD [18–20]. For example, parcellation of structural magnetic resonance imaging (MRI) data has been used in many studies as brain structure changes greatly in AD [21–24]. Further, in two studies, we showed that T1-weighted MRI (structural MRI; sMRI) can be used in classification of AD and MCI. Indeed, the majority of early studies looking at classification of AD and HC was done on sMRI [22]. This is mostly due to costs and accessibility of sMRI data [23].

While structural neuroimaging has shown some success in early detection of AD, functional neuroimaging has proven to be a stronger candidate [25–27]. Functional MRI (fMRI) allows for the examination of brain functioning while a patient is performing a cognitive task. This technique is especially well suited to identifying changes in brain functioning before significant impairments can be detected on standard neuropsychological tests, and as such is sensitive to early identification of the disease processes [28,29]. While fMRI requires participants to perform a task, resting-state fMRI (rs-fMRI) is capable of measuring the spontaneous fluctuations of brain activity without any task, hence it is less sensitive to individual cognitive abilities [30–32].

One important feature of rs-fMRI is the ability to measure functional connectivity changes [33,34], which has been shown to be a prevalent change in AD [35–38]. Furthermore, it is shown that the increased severity of cognitive impairment is associated with increasing alteration in connectivity patterns, suggesting that disruptions in functional connectivity may contribute to cognitive dysfunction and may represent a potential biomarker of impaired cognitive ability in MCI. In particular, research has highlighted that longitudinal alterations of functional connectivity are more profound in earlier stages as opposed to later stages of the disease [39]. Therefore, analysis of functional connectivity can provide an excellent opportunity in identification of early states of AD.

As functional connectively analysis inherently relies of networks of activity, researchers have used graph theory measures to investigate the global, as well as local, characteristics of different brain areas [40–43] Click or tap here to enter text. This method has been used successfully in a wide range of application in both healthy participants and patients [44] Click or tap here to enter text. such as depression [45,46] Click or tap here to enter text., Parkinson’s disease [47] Click or tap here to enter text., as well as AD [48] Click or tap here to enter text. Graph theories provides us with a way to study AD [48–53] and comprehensively compare functional connectivity organization of the brain between patients and controls [43–45] Click or tap here to enter text. and importantly between different stages of AD [54,55] Click or tap here to enter text. This method can also unveil compensatory mechanisms, thus revealing brain functional differences in participants with comparable level of cognitive ability [56–59] Click or tap here to enter text.

Graph theory analysis of rs-fMRI data, however, leads to a large number of parameters. Therefore, to reduce computational complexity, it is essential to select an optimal subset of features that can lead to high discrimination accuracy [60,61]. Feature selection is particularly complicated due to the non-linear nature of classification methods: more parameters do not necessarily lead to better performance, and there is also a dependency of parameters [62,63]. Therefore, it is extremely important to utilize a suitable optimization method that can deal with nonlinear high-dimensional search spaces.

Evolutionary algorithms (EA) are biologically-inspired algorithms that are extremely effective in optimization algorithms with large search spaces [64–66]. These methods, in contrast with many other search methods such as complete search, greedy search, heuristic search and random search [67,68], do not suffer from stagnation in local optima and/or high computational cost [69,70]. Feature selection has been used to improve the quality of the feature set in many machine learning tasks, such as classification, clustering and time-series prediction [71]. Classification and time-series prediction are particularly relevant to many neurodegenerative diseases: classification can be used in identification of those with brain damage [72,73] and time-series prediction can be used in estimation of disease progression [74,75].

EA has been used in characterization and diagnosis of AD [76–78]. Such methods have achieved reasonably high accuracy in classification of AD and HC (70–95%). They, however, have been unsuccessful in classification of the MCI patients [79]. Therefore, in this study, we devised a method that achieves higher accuracy in the classification of HC and EMCI participants compared to the past-published research. We used MRI and rs-fMRI data of a group of healthy participants and those with EMCI. We applied graph theory to extract a collection of 1155 parameters. This data is then given to five different EA methods to select an optimum subset of parameters. These selected parameters are subsequently given to an artificial neural network to classify the data into two groups of HC and EMCI. We aimed at identifying the most suitable method of optimization based on accuracy and training time, as well as identifying the most informative parameters.

## Methods

### Participants

Data used in the preparation of this article were obtained from the Alzheimer’s Disease Neuroimaging Initiative (ADNI) database (adni.loni.usc.edu). The ADNI was launched in 2003 as a public-private partnership, led by Principal Investigator Michael W. Weiner, MD. The primary goal of ADNI has been to test whether serial magnetic resonance imaging (MRI), positron emission tomography (PET), other biological markers, and clinical and neuropsychological assessment can be combined to measure the progression of mild cognitive impairment (MCI) and early Alzheimer’s disease (AD).

Data for 140 participants were extracted from the ADNI [80–82]. See Table 1 for the details of the data. EMCI participants had no other neurodegenerative diseases except MCI. The EMCI participants were recruited with memory function approximately 1.0 SD below expected education adjusted norms [83]. HC subjects had no history of cognitive impairment, head injury, major psychiatric disease, or stroke.

**Table 1:** Demographics of the data for participants included in this study.

	EMCI	HC	*P*
n	68	72	
Female (n [%])	38 [55.88]	38 [52.77]	
Age (mean [SD])	71.73 [7.80]	69.97 [5.60]	0.297
MMSE (mean [SD])	28.61 [1.60]	28.40 [4.60]	0.302
CDR	0.5 or 1	0	< 0.001

notes: CDR: Clinical dementia rating, MMSE: Mini-mental state exam, HC: Healthy control, EMCI: Early mild cognitive impairment.

### Proposed method

**Fig 1:**
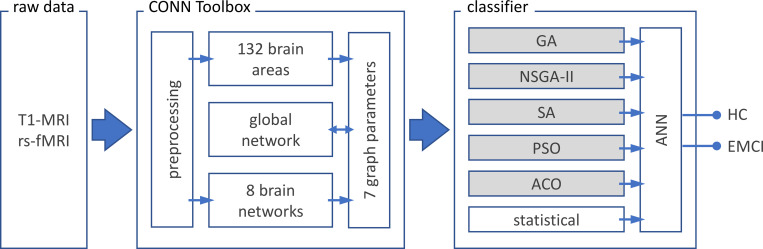
Procedure of the proposed method. T1-MRI (sMRI) and resting-state fMRI (rs-fMRI) data of healthy participants (HC; n = 72) and patients with early mild cognitive impairment (EMCI; n = 68) are extracted from ADNI database ^82^. Preprocessing, parcellation of brain area (132 regions based on AAL and Harvard-Oxford atlas) and extraction of the functional connectivity (8 network parameters with a total of 32 nodes), as well as the 7 graph parameters are done using CONN toolbox ^84^. Subsequently the global network is calculated based on the network parameters. The 1155 ([132 brain regions + 32 nodes of brain networks + 1 global network] × 7 graph parameters) extracted parameters are given to one of the optimization methods to select the best subset of parameters that lead to best classification method. Optimization methods consisted of five evolutionary algorithms (boxes with grey shading) and one statistical algorithm. The outputs of these methods are given to an artificial neural network (ANN) with two hidden layers to classify the data into HC and EMCI. AAL: Automated anatomical atlas; GA: Genetic algorithm; NSGA-II: Nondominated sorting genetic algorithm II; ACO: Ant colony optimization; SA: Simulated annealing; PSO: Particle swarm optimization; seven graph features: Degree centrality, betweenness centrality, path length, clustering coefficient, local efficiency, cost and global efficiency.

sMRI and rs-fMRI data was extracted from the ADNI database [82]. The data is given to CONN toolbox [84] in MATLAB v2018 (MathWorks, California, US). CONN is a tool for preprocessing, processing, and analysis of functional connectivity data. Preprocessing consisted of reducing subject motion, image distortions, and magnetic field inhomogeneity effects and application of denoising methods for reduction of physiological effects and other sources of noise. The processing stage consisted of extraction of functional connectivity and graph theory measures. In this stage, through two pipelines, a collection of 1155 parameters are extracted (see below) [84,85]. These parameters are then given to one of the dimension reduction methods (five EA and one statistical method) to select a subset of features. The selected features are finally given to an artificial neural network to classify the data into two categories of HC and EMCI. The classification method was performed via a 90/10 split; 90% of the data was used for the training and 10% of the data was used for validation. See Fig 1 for the summary of the procedure of the method.

### Data acquisition and processing

Brain structural sMRI data with 256×256×170 voxels and 1×1×1 mm^3^ voxel size were extracted for all subjects. MRI data preprocessing steps consisted of non-uniformity correction, segmentation into grey matter, white matter and cerebrospinal fluid (CSF) and spatial normalization to MNI space.

Rs-fMRI data were obtained using an echo-planar imaging sequence on a 3T Philips MRI scanner. Acquisition parameters were: 140 time points, repetition time (TR) = 3000 ms, echo time (TE) = 30 ms, flip angle = 80°, number of slices = 48, slice thickness = 3.3 mm, spatial resolution = 3×3×3 mm^3^ and in plane matrix = 64×64. FMRI images preprocessing steps consisted of motion correction, slice timing correction, spatial normalization to MNI space, low frequency filtering to keep only (0.01–0.1 Hz) fluctuations.

CONN toolbox [84,85] is used to process the sMRI and rs-fMRI data. The output of this toolbox is 1155 values consisting of: (a) 132 distinct brain areas according to Automated Anatomical Labeling (AAL) and Harvard-Oxford atlases, (b) eight brain networks containing 32 nodes and (c) a global network parameter that is the average of seven graph parameters [86–88]. All these values are multiplied by seven graph parameters, see below. See supplementary data for details of these parameters. The sMRI images were used to register the functional images and improve the analysis of the rs-fMRI data.

### Functional connectivity

Functional connectivity, also called “resting state” connectivity, is a measure for the temporal correlations among the blood-oxygen-level-dependent (BOLD) signal fluctuations in different brain areas [89–91]. The functional connectivity matrix is the correlation, covariance, or the mutual information between the fMRI time series of every two brain regions, which is stored in an *n*×*n* matrix for each participant, where n is the number of brain regions obtained by atlas parcellation [91]. To extract functional connectivity between different brain areas we used Pearson correlation coefficients formula as following [84,92]:

$$r(x)=\frac{\int S(x,t)R(t)dt}{{(\int R^{2}(t)dt\int S^{2}(x,t)dt)}^{\frac{1}{2}}}$$

$$Z(x)={tanh}^{-1}(r(x)),$$

where *S* is the BOLD time series at each voxel (for simplicity all-time series are considered central to zero means), *R* is the average BOLD time series within an ROI, *r* is the spatial map of Pearson correlation coefficients, and *Z* is the seed-based correlations (SBC) map of Fisher-transformed correlation coefficients for this ROI [93].

### Graph parameters

We used the graph theory technique to study topological features of functional connectivity graphs across multiple regions of the brain [86–94]. Graph nodes represented brain regions and edges represented interregional resting-state functional connectivity. The graph measurements in all of the ROIs are defended using nodes = ROIs, and edges = suprathreshold connections. For each subject, graph adjacency matrix A is computed by thresholding the associated ROI to-ROI Correlation (RRC) matrix r by an absolute (e.g., z>0.5) or relative (e.g., highest 10%) threshold. Then, from the resulting graphs, some measurements can be computed addressing topological properties of each ROI within the graph as well as of the entire network of ROIs. The adjacency matrix is employed for estimating common features of graphs including (1) *degree centrality* (the number of edges that connect a node to the rest of the network) (2) *betweenness centrality* (the proportion of shortest paths between all node pairs in the network that pass through a given index node), (3) *average path length* (the average distance from each node to any other node), (4) *clustering coefficient* (the proportion of ROIs that have connectivity with a particular ROI that also have connectivity with each other), (5) *cost* (the ratio of the existing number of edges to the number of all possible edges in the network), (6) *local efficiency* (the network ability in transmitting information at the local level), (7) *global efficiency* (the average inverse shortest path length in the network; this parameter is inversely related to the path length) [95].

### Dimension reduction methods

We used five EA to select the most efficient set number of features. These algorithms are as follows:

*Genetic algorithm (GA)*: GA is one of the most advanced algorithms for feature selection [96]. This algorithm is based on the mechanics of natural genetics and biological evolution for finding the optimum solution. It consists of five steps: selection of initial population, evaluation of fitness function, pseudo-random selection, crossover, and mutation [97]. For further information refer to supplementary Methods section. Single point, double point, and uniform crossover methods are used to generate new individuals. In this study we used 0.3 and 0.1 as mutation percentage and mutation rate, respectively; 20 members per population, crossover percentage was 14 with 8 as selection pressure [74,98].

*Nondominated sorting genetic algorithm II (NSGA-II)*: NSGA is a method to solve multi-objective optimization problems to capture a number of solutions simultaneously [99]. All the operators in GA are also used here. NSGA-II uses binary features to fill a mating poll. Nondomination and crowding distance are used to sort the new members. For further information refer to supplementary Methods section. In this study the mutation percentage and mutation rate were set to 0.4 and 0.1, respectively; population size was 25, and crossover percentage was 14.

*Ant colony optimization algorithm (ACO)*: ACO is a metaheuristic optimization method based on the behavior of ants [100]. This algorithm consists of four steps: initialization, creation of ant solutions (a set of ants build a solution to the problem being solved using pheromones values and other information), local search (improvement of the created solution by ants), and global pheromone update (update in pheromone variables based on search action followed by ants) [101]. ACO requires a problem to be described as a graph: nodes represent features and edges indicate which features should be selected for the next generation. In features selection, the ACO tries to find the best solutions using prior information from previous iterations. The search for the optimal feature subset consists of an ant traveling through the graph with a minimum number of nodes required for satisfaction of stopping criterion [102]. For further information refer to supplementary Methods section. We used 10, 0.05, 1, 1 and 1 for the number of ants, evaporation rate, initial weight, exponential weight, and heuristic weight, respectively.

*Simulated annealing (SA)*: SA is a stochastic search algorithm, which is particularly useful in large-scale linear regression models [103]. In this algorithm, the new feature subset is selected entirely at random based on the current state. After an adequate number of iterations, a dataset can be created to quantify the difference in performance with and without each predictor [104,105]. For further information refer to supplementary Methods section. We set initial temperature and temperature reduction rate with 10 and 0.99, respectively.

*Particle swarm optimization (PSO)*: PSO is a stochastic optimization method based on the behavior of swarming animals such as birds and fish. Each member finds optimal regions of the search space by coordinating with other members in the population. In this method, each possible solution is represented as a particle with a certain position and velocity moving through the search space [106–108]. Particles move based on cognitive parameter (defining the degree of acceleration towards the particle’s individual local best position, and global parameter (defining the acceleration towards the global best position). The overall rate of change is defined by an inertia parameter. For further information refer to supplementary Methods section. In this paper simulation we use 20 as the warm size, cognitive and social parameters were set to 1.5 and inertia as 0.72.

*Statistical approach*: To create a baseline to compare dimension reduction methods based on evolutionary algorithms, we also used the statistical approach to select the features based on the statistical difference between the two groups. We compared the 1155 parameters using two independent-sample t-test analyses. Subsequently we selected the parameters based on their sorted *p* values.

### Classification method

For classification of EMCI and HC we used a multi-layer perceptron artificial neural network (ANN) with two fully-connected hidden layers with 10 nodes each. Classification method was performed via a 10-fold cross-validation. We used Levenberg-Marquardt Back propagation (LMBP) algorithm for training [109–111] and mean square error as a measure of performance. The LMBP has three steps: (1) propagate the input forward through the network; (2) propagate the sensitivities backward through the network from the last layer to the first layer; and finally (3) update the weights and biases using Newton’s computational method [109]. In the LMBP algorithm the performance index *F*(*x*) is formulated as:

$$F(x)=e^{T}(x)e(x),$$

where *e* is vector of network error, and *x* is the vector matrix of network weights and biases. The network weights are updated using the Hessian matrix and its gradient:

$$x_{k+1}=x_{k}-{\left(J^{T}J+\mu I\right)}^{-1}J^{T}e=x_{k}-{\left(H+\mu I\right)}^{-1}G,$$

Where *J* represent Jacobian matrix. The Hessian matrix *H* and its gradient *G* are calculated using:

$$H=J^{T}J$$

$$G=J^{T}e,$$

where the Jacobian matrix is calculated by:

$$J=S^{m}a^{m-1},$$

where *a*^*m*−1^ is the output of the (*m*−1)th layer of the network, and *S*^*m*^ is the sensitivity of *F*(*x*) to changes in the network input element in the *m*th layer and is calculated by:

$$S^{m}=F^{m}(n^{m})(w^{m+1})S^{m+1},$$

where *w*^*m*+1^ represents the neuron weight at (*m*+1)th layer, and *n* is the network input [109].

## Results

**Fig 2:**
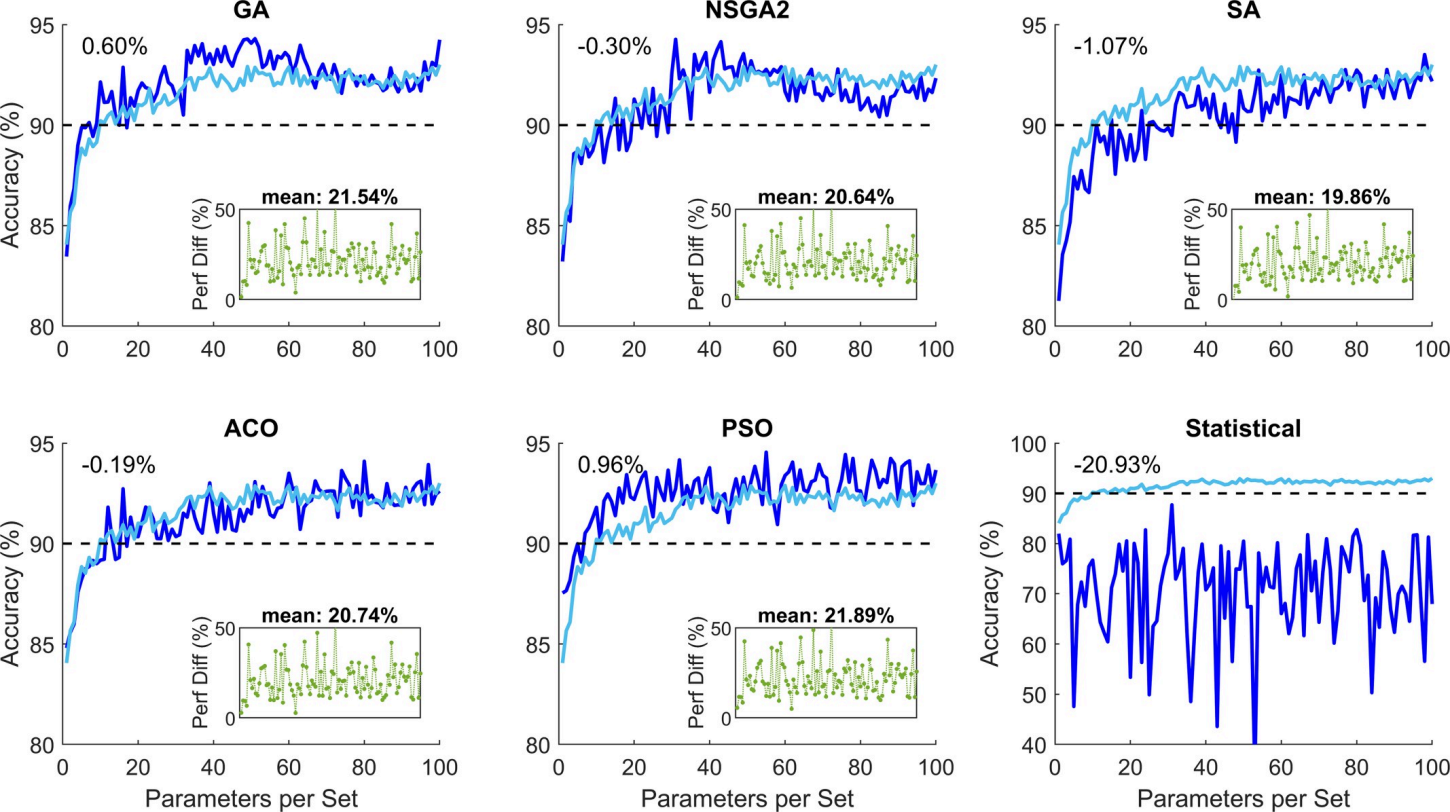
Classification performance of the five evolutionary algorithm (EA) methods and the statistical method for parameter subsets with 1 to 100 elements. The light blue color shows the average of the five EV algorithms. The number on the top left-hand corner represents the difference between the relevant plot and the mean performance of the EA methods. The green plot subplot in each panel represents superiority of the relevant EA as compared to the statistical method for different 100 subsets. The percentage value above the subplot shows the mean superior performance for the 100 subsets compared to the statistical method. These plots show that the EA performed significantly better than the statistical method. GA: Genetic algorithm; NSGA2: Nondominated sorting genetic algorithm II; ACO: Ant colony optimization; SA: Simulated annealing; PSO: Particle swarm optimization.

The preprocessing and processing of the data was successful. We extracted 1155 graph parameters per participant (see S1–S11 Figs). This data was used for the data optimization step. Using the five EA optimization methods and the statistical method, we investigated the performance of the classification for different numbers of subset of parameters. Fig 2 shows the performance of these methods for 100 subsets of parameters with 1 to 100 parameters. These plots are created based on 200 repetitions of the EA algorithms. To investigate the performance of the algorithms with more repetitions, we ran the same algorithms with 500 repetitions. These simulations showed no major improvement of increased repetition (maximum 0.84% improvement; see S11 Fig).

**Fig 3:**
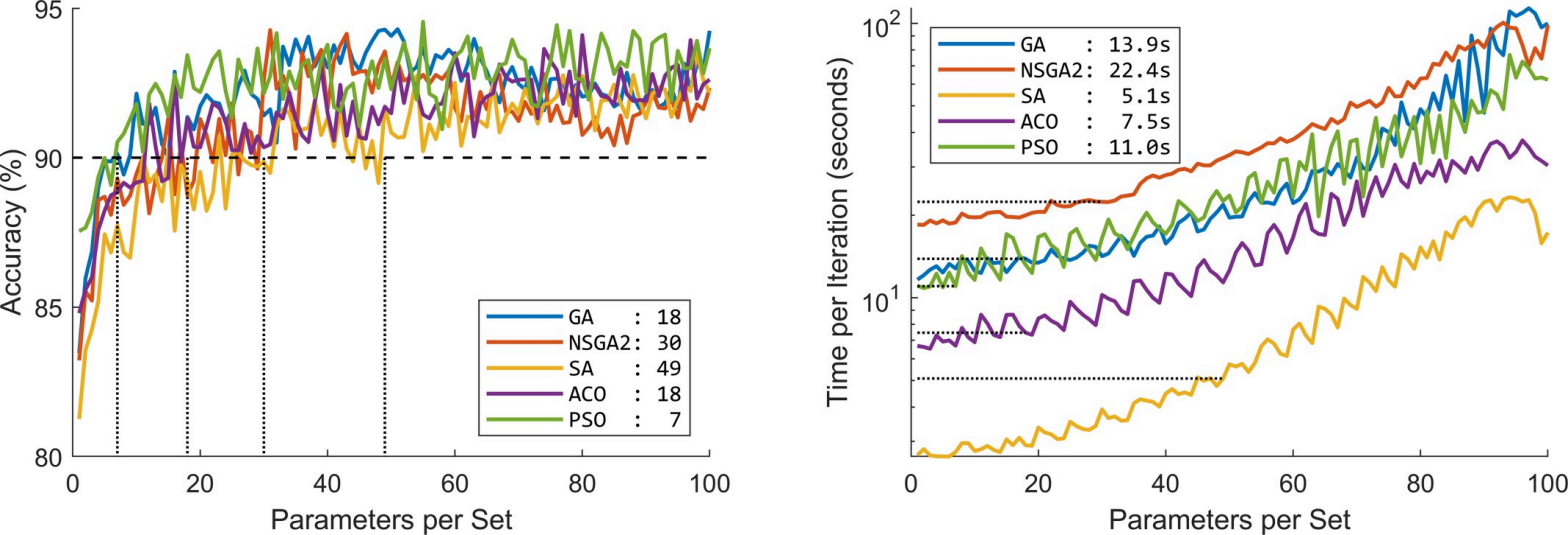
Performance of the five evolutionary algorithms (EA) in terms of (a) percentage accuracy and (b) optimization speed. The values in the legend of panel (a) show the minimum number of parameters required to achieve minimum 90% accuracy. The values in the legend of panel (b) show the minimum optimization speed to achieve minimum 90% accuracy based on panel (a). GA: Genetic algorithm; NSGA2: Nondominated sorting genetic algorithm II; ACO: Ant colony optimization; SA: Simulated annealing; PSO: Particle swarm optimization.

A threshold of 90% was chosen as the desired performance accuracy. Statistical modeling performance constantly less than this threshold. The five EA methods achieved this performance with varying number of parameters. Fig 3 shows the accuracy percentage and the optimization speed of the five EA methods.

There is growing body of literature showing gender differences. It has been shown that women are more likely to suffer from AD. Therefore, to investigate whether our analysis method performs better on a particular gender or not, we split the data into two groups of female and male participants. Our analysis showed that there is no meaningful difference between the two groups (see S2 Table).

**Fig 4:**
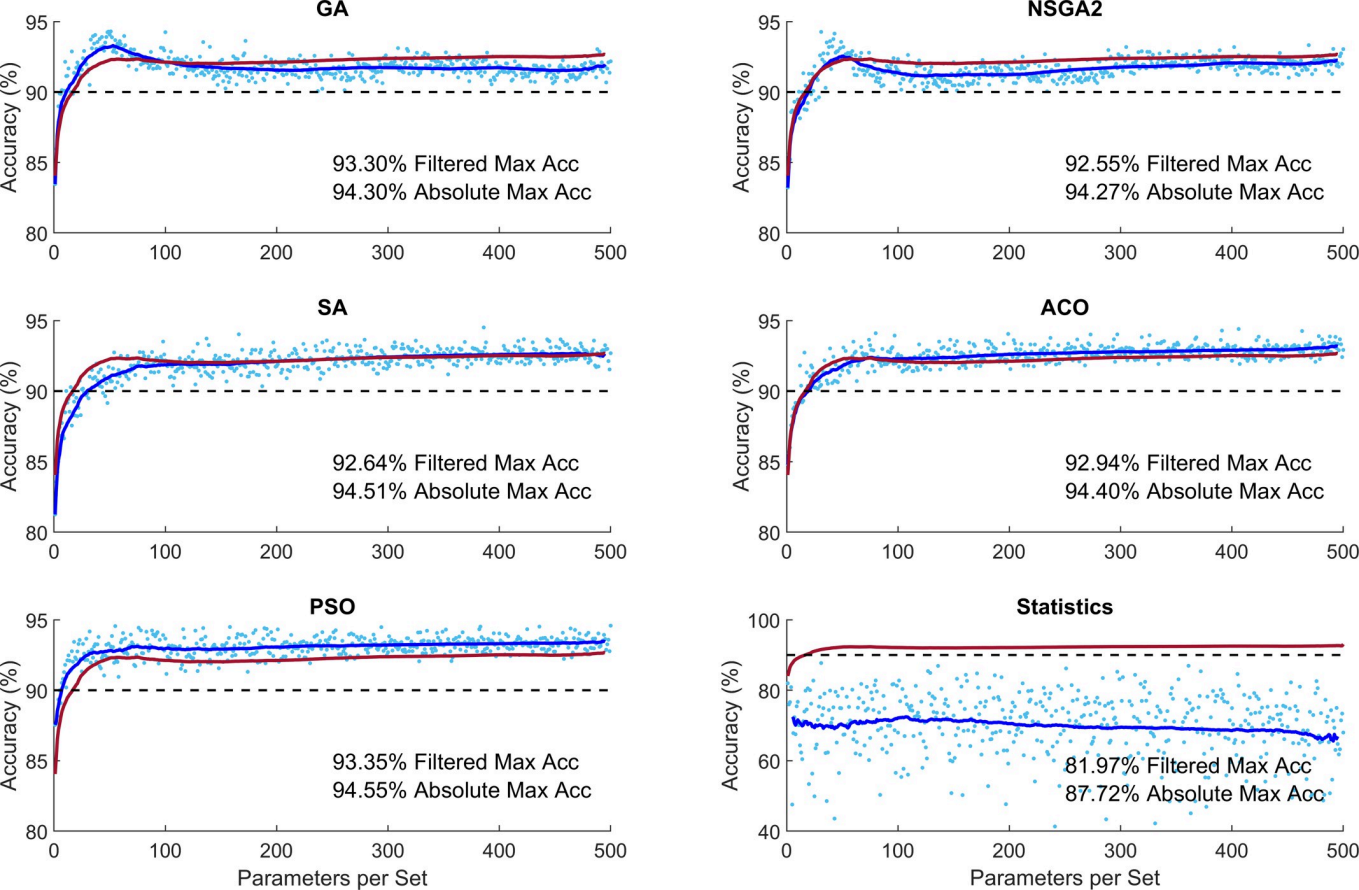
Performance of different optimization methods for increased number of parameters per subset. The light blue dots indicate the performance of algorithms for each subset of parameters. The dark blue curve shows the moving average of the samples with window of ±20 points (Filtered Data). The red curve shows the mean performance of the five evolutionary algorithms. GA: Genetic algorithm; NSGA2: Nondominated sorting genetic algorithm II; ACO: Ant colony optimization; SA: Simulated annealing; PSO: Particle swarm optimization.

To investigate whether increasing number of parameters would increase performance, we performed similar simulations with maximum 500 parameters in each subset. This analysis showed that the performance of the optimization methods plateaus without significant increase from 100 parameters (Fig 4). This figure shows that performance of the optimization methods was between 92.55–93.35% and 94.27–94.55% for filtered and absolute accuracy, respectively. These accuracy percentages are significantly higher than 81.97% and 87.72% for filtered and absolute accuracy in the statistical classification condition.

**Fig 5:**
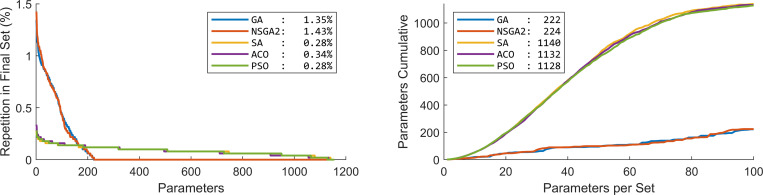
Distribution of different parameters over the 100 subsets of parameters. (a) Percentage of presence of the 1155 parameters. In the Statistical method, which is not present in the plot, the first parameter was repeated in all the 100 subsets. Numbers in the legend show the percentage repetition of the most repeated parameter. (b) Cumulative number of unique parameters over the 100 subsets of parameters. This plot shows that GA and NSGA2 concentrated on a small number of parameters, while the SA, ACO and PSO selected a more diverse range of parameters in the optimization. Numbers in the legend show the number of utilized parameters in the final solution of the 100 subsets of parameters. GA: Genetic algorithm; NSGA2: Nondominated sorting genetic algorithm II; ACO: Ant colony optimization; SA: Simulated annealing; PSO: Particle swarm optimization.

To investigate the contribution of different parameters in the optimization of classification we looked at the distribution of parameters in the 100 subsets calculated above (Fig 5). GA and NSGA showed that the majority of the subsets consisted of repeated parameters: out of the 1155 parameters only about 200 of the parameters were selected in the 100 subsets. SA, ACO and PSO, on the other hand, showed a more diverse selection of parameters: almost all the parameters appeared in at least one of the 100 subsets.

To identify the parameters that are most predominantly involved in classification of HC and EMCI, we extracted the list of the five most indicative brain regions and networks, Table 2. These are selected based on the total number of times that they appeared in the 100 simulations using the five EA’s.

**Table 2:** Summary of the five most indicative brain areas and networks in the classification of healthy (HC) and early mild cognitive impairment (EMCI).

	Brain Area		Brain Networks	
Method	Area	Graph Param.	Network	Graph Param.
GA	SFG	Local Efficiency	Global Network	Global Efficiency
	Insular Cortex	Local Efficiency	Global Network	Local Efficiency
	Frontal Pole	Degree Centrality	Global Network	Clustering Coefficient
	Middle Frontal Gyrus	Betweenness Centrality	Global Network	Average Path Length
	Inferior Frontal Gyrus; pars triangularis	Clustering Coefficient	Global Network	Betweenness Centrality
NSGA-II	Insular Cortex	Local Efficiency	Global Network	Betweenness Centrality
	SFG	Local Efficiency	Global Network	Local Efficiency
	Frontal Pole	Degree Centrality	Global Network	Cost
	Middle Frontal Gyrus	Betweenness Centrality	Global Network	Clustering Coefficient
	Precentral Gyrus	Local Efficiency	Global Network	Global Efficiency
SA	SFG	Local Efficiency	Language–IFG	Degree Centrality
	IFG; pars triangularis	Degree Centrality	Visual–Occipital	Global Efficiency
	Lingual Gyrus	Betweenness Centrality	Visual–Lateral	Average Path Length
	Thalamus	Cost	Dorsal Attention–FEF	Betweenness Centrality
	MTG; temporooccipital part	Average Path Length	Language–pSTG	Degree Centrality
ACO	Occipital Pole	Local Efficiency	Fronto-parietal–PPC	Degree Centrality
	SFG	Global Efficiency	Dorsal Attention–FEF	Local Efficiency
	Middle Frontal Gyrus	Global Efficiency	Visual–Occipital	Cost
	Inferior Temporal Gyrus; posterior division	Degree Centrality	Default Mode–LP	Cost
	Intracalcarine Cortex	Betweenness Centrality	Dorsal Attention–FEF	Clustering Coefficient
PSO	Occipital Pole	Local Efficiency	Dorsal Attention–FEF	Local Efficiency
	SFG	Local Efficiency	Visual–Medial	Global Efficiency
	Frontal Medial Cortex	Betweenness Centrality	Dorsal Attention–FEF	Local Efficiency
	Supplementary Motor Cortex	Local Efficiency	Language–pSTG	Clustering Coefficient
	Lingual Gyrus	Betweenness Centrality	Salience–Anterior Insula	Degree Centrality

Notes: FEF: Frontal-eye-field, IFG: Inferior frontal gyrus, LP: Lateral parietal, PPC: Posterior parietal cortex, MTG: Middle temporal gyrus, pSTG: Posterior superior temporal gyrus, SFG: Superior frontal gyrus.

## Discussions

Using CONN toolbox, we extracted 1155 graph parameters from rs-fMRI data. The optimization methods showed superior performance over statistical analysis (average 20.93% superiority). The performance of the EA algorithms did not differ greatly (range 92.55–93.35% and 94.27–94.55% for filtered and absolute accuracy, respectively) with PSO performing the best (mean 0.96% superior performance) and SA performing the worst (mean 1.07% inferior performance), (Fig 2). The minimum number of required parameters to guarantee at least 90% accuracy differed quite greatly across the methods (PSO and SA requiring 7 and 49 parameters, respectively). The processing time to achieve at least 90% accuracy also differed across the EA methods (SA and NSGA2 taking 5.1s and 22.4s per optimization) (Fig 3). Increased number of parameters per subset did not increase the performance accuracy of the methods greatly (Fig 4).

Classification of data into AD and HC has been investigated extensively. Many methods have been developed using different modalities of biomarkers. Some of these studies achieved accuracies greater than 90% [112]. Classification of earlier stages of AD, however, has been more challenging; only a handful of studies have achieved accuracy higher than 90%, Table 3. The majority of these studies implemented convolutional and deep neural networks that require extended training and testing durations with many input data. For example, Payan et al. (2015) applied convolutional neural networks (CNN) on a collection of 755 HC and 755 MCI and achieved accuracy of 92.1% [113]. Similarly, Wang et al. (2019) applied deep neural networks to 209 HC and 384 MCI data and achieved accuracy of 98.4% [114] (see also [115–118]). Our method achieved an accuracy of 94.55%. To the best of our knowledge, between all the studies published to date, this accuracy level is the second highest accuracy after Wang et al (2019) [114].

**Table 3:** Summary of the studies aiming at categorization of healthy (HC) and mild cognitive impairment (MCI) using different biomarkers and classification methods. Only best performance of each study is reported for each group of participants and classification method. Further details of the following studies are in S1 Table.

				HC	MCI		
Study ↑	Cit.	Method	Modalities	n	Cat.	n	Acc%
Wolz et al (2011)	[119]	LDA	MRI	231	sMCI	238	68
					cMCI	167	84
Zhang et al (2011)	[120]	SVM	MRI+FDG-PET+ CSF	231	SMCI	238	82
		LDA			PMCI	167	84
Liu et al (2012)	[121]	SRC	MRI	229	MCI	225	87.8
Gray et al (2013)	[122]	RF	MRI+PET+CSF+genetic	35	MCI	75	75
Liu et al (2013)	[123]	SVM + LLE	MRI	137	sMCI	92	69
					cMCI	97	81
Wee et al (2013)	[124]	SVM	MRI	200	MCI	200	83.7
Guerrero et al (2014)	[125]	SVM	MRI	134	EMCI	229	65
			MRI	175	cMCI	116	82
Payan & Montana (2015)	[113]	CNN	MRI	755	MCI	755	92.1
Prasad et al (2015)	[127]	SVM	DWI	50	EMCI	74	59.2
					LMCI	38	62.8
Suk et al (2015)	[127]	DNN	MRI+PET	52	MCI	99	90.7
Shakeri et al (2016)	[128]	DNN	MRI	150	EMCI	160	56
			MRI		LMCI	160	59
Aderghal, Benois-Pineau et al (2017)	[129]	CNN	MRI	228	MCI	399	66.2
Aderghal, Boissenin et al (2017)	[130]	CNN	MRI	228	MCI	399	66
Billones et al (2017)	[115]	CNN	MRI	300	MCI	300	91.7
Guo et al (2017)	[131]	SVM	rs-fMRI	28	EMCI	32	72.8
					LMCI	32	78.6
Korolev et al (2017)	[132]	CNN	MRI	61	LMCI	43	63
					EMCI	77	56
Wang et al (2017)	[116]	CNN	MRI	229	MCI	400	90.6
Li & Liu (2018)	[133]	CNN	MRI	229	MCI	403	73.8
Qiu et al (2018)	[117]	CNN	MRI+MMSE+ LM	303	MCI	83	90.9
Senanayake et al (2018)	[134]	CNN	MRI+NM	161	MCI	193	75
Altaf et al (2018)	[135]	SVM	MRI	90	MCI	105	79.8
		Ensemble	MRI		MCI		75
		KNN	MRI		MCI		75
		Tree	MRI		MCI		78
		SVM	clinical+MRI		MCI		83
		Ensemble	clinical+MRI		MCI		82
		KNN	clinical+MRI		MCI		86
		Tree	clinical+MRI		MCI		80
Forouzannezhad et al (2018)	[118]	SVM	MRI	248	EMCI	296	73.1
			MRI		LMCI	193	63
			PET		LMCI		73.6
			PET+MRI		LMCI		76.9
			PET+MRI		EMCI		75.6
			PET+MRI+NTS		LMCI		91.9
			PET+MRI+NTS		EMCI		81.1
Hosseini Asl et al (2018)	[136]	CNN	MRI	70	MCI	70	94
Jie, Liu, Shen et al (2018)	[137]	SVM	rs-fMRI	50	EMCI	56	78.3
Jie, Liu, Zhang et al (2018)	[138]	SVM	rs-fMRI	50	MCI	99	82.6
Raeper et al (2018)	[118]	SVM + LDA	MRI	42	EMCI	42	80.9
Basaia et al (2019)	[140]	CNN	MRI	407	cMCI	280	87.1
					sMCI	533	76.1
Forouzannezhad et al (2019)	[141]	DNN	MRI	248	EMCI	296	61.1
			MRI		LMCI	193	64.1
			PET		EMCI		58.2
			PET		LMCI		66
			MRI+PET		EMCI		68
			MRI+PET		LMCI		71.7
			MRI+PET+NTS		EMCI		84
			MRI+PET+NTS		LMCI		84.1
Wang et al (2019)	[114]	DNN	MRI	209	MCI	384	98.4
Wee et al (2019)	[142]	CNN	MRI	300	LMCI	208	69.3
					EMCI	314	51.8
				242	MCI	415	67.6
Kam et al (2020)	[143]	CNN	rs-fMRI	48	EMCI	49	76.1
Fang et al (2020)	[144]	GDCA	MRI+PET	251	EMCI		79.2
Forouzannezhad et al (2020)	[145]	GP	MRI	248	EMCI	296	75.9
			MRI		LMCI	193	62.1
			MRI+PET		EMCI		75.9
			MRI+PET		LMCI		78.1
			MRI+PET+DI		EMCI		78.8
			MRI+PET+DI		LMCI		79.8
			PET		LMCI		76.1
Jiang et al (2020)	[146]	CNN	MRI	50	EMCI	70	89.4
Kang et al (2020)	[147]	CNN	DTI	50	EMCI	70	71.7
		CNN	MRI		EMCI		73.3
			DTI+MRI		EMCI		94.2
Yang et al (2021)	[148]	SVM	rs-fMRI	29	EMCI	29	82.8
					LMCI	18	87.2
**our method**		**EA + ANN**	**rs-fMRI**	**68**	**EMCI**	**72**	**94.5**

Notes: ↑ table sorted based on the year of publication. Acc: Classification accuracy percentage between MCI and HC groups; ANN: Artificial neural networks; Cat.: Category of MCI; Cit.: Citation; cMCI: MCI converted to AD; CNN: Convolutional neural networks; DI: Demographic information; DNN: Deep neural network; DTI: Diffusion tensor imaging; DWI: Diffusion-weighted imaging; EA: Evolutionary algorithms; EMCI: Early-MCI; GDCA: Gaussian discriminative component analysis; GP: Gaussian process; KNN: K nearest neighbors; LDA: Linear discriminative analysis; LLE: Locally linear embedding; LM: Logical memory; LMCI: Late-MCI; NTS: Neuropsychological test scores; MMSE: Mini-mental state examination (MMSE); NM: Neuropsychological measures; PET: Positron emission therapy; rs-fMRI: Resting-state fMRI; sMCI: Stable MCI; NM: Neuropsychological measures; SRC: Sparse representation-based classifier; SVM: Support vector machine.

Research has shown that having a combination of information from different modalities supports higher classification accuracies. For example, Forouzannezhad et al. (2018) showed that a combination of PET, MRI and neuropsychological test scores (NTS) can improve performance by more than 20% as compared to only PET or MRI [118]. In another study, Kang et al. (2020) showed that a combination of diffusion tensor imaging (DTI) and MRI can improve accuracy by more than 20% as compared to DTI and MRI alone [147]. Our analysis, while achieving superior accuracy compared to a majority of the prior methods, was based on one biomarker of MRI, which has a lower computational complexity than multi-modality data.

Interpretability of the selected features is one advantage of the application of evolutionary algorithms as the basis of the optimization algorithm. This is in contrast with algorithms based on CNN or deep neural networks (DNN) that are mostly considered as black boxes [149]. Although research has shown some progress in better understanding the link between the features used by the system and the prediction itself in CNN and DNN, such methods remain difficult to verify [150,151]. This has reduced trust in the internal functionality and reliability of such systems in clinical settings [152]. Our suggested method clearly selects features based on activity of distinct brain areas, which are easy to interpret and understand [78–153]. This can inform future research by bringing the focus to brain areas and the link between brain areas that are more affected by mild cognitive impairment.

Our analysis showed that dorsal attention network is altered in EMCI, confirming past literature [154–156]. Dorsal attention network in addition to the ventral attention network form the human attention system [157]. The dorsal attention network employs dorsal fronto-parietal areas, including intraparietal sulcus (IPS) and frontal eye fields (FEF). It is involved in mediation of goal-directed process and selection for stimuli and response. Specifically, our data highlighted the role of the FEF in the dorsal attention network. This is in line with past literature showing the role of FEF in cognitive decline [158]. Our data also revealed the importance of superior frontal gyrus (SFG) in cognitive decline [159,160]. SFG is thought to contribute to higher cognitive functions and particularly to working memory (WM) [161]. Additionally, SFG interconnects multiple brain areas that are involved in a diverse range of cognitive tasks such as cognitive control and motor behavior [162].

In terms of graph parameters, our results showed importance of local efficiency, betweenness centrality and degree centrality in classification of EMCI and HC. Local efficiency is a parameter for the transformation of information in a part of the network. This parameter indicates the efficiency between two nodes and represents the efficiency in exchange of information through a network edge [87,163]. Reduction of this parameter has been linked with cognitive decline in past literature [164]. Betweenness centrality for any given node (vertex) measures the number of shortest paths between pairs of other nodes that pass through this node, reflecting how efficiently the network exchanges the information at the global level. Betweenness centrality is high for nodes that are located on many short paths in the network and low for nodes that do not participate in many short paths [164]. Finally, degree centrality reflects the number of instantaneous functional connections between a region and the rest of the brain within the entire connectivity matrix of the brain. It can assess how much a node influences the entire brain and integrates information across functionally segregated brain regions [165] (see also [166]). Our data showed that changes in these parameters can effectively contribute in classification of early-MCI patients from healthy controls.

We implemented five of the most common evolutionary algorithms. They showed similar overall optimization performance ranging between 92.55–93.35% and 94.27–94.55% for filtered and absolute accuracy, respectively. They, however, differed in optimization curve, optimization time and diversity of the selected features. PSO could guarantee a 90% accuracy with only 7 features. SA on the other hand required 49 features to guarantee a 90% accuracy. Although SA required more features to guarantee a 90% accuracy, it was the fastest optimization algorithm with only 5.1s for 49 features. NSGA-II on the other hand, required 22.4s to guarantee a 90% accuracy. These show the diversity of the algorithms and their suitability in different applications requiring highest accuracy, least number of features or fastest optimization time [71,76,167].

One distinct characteristic of GA and NSGA-II was the more focused search amongst features as compared to the other methods. GA and NSGA-II selected 222 and 224 distinct features in the first 100 parameter sets, respectively, while the other methods covered almost the whole collection of features, covering more than 97.6%. Notably GA and NSGA-II showed “curse of dimensionality” (also known as “peaking phenomenon”) with optimal number of features around 50 parameters [168–171]. Therefore, perhaps the features selected by GA and NSGA-II are more indicative of distinct characteristics of the differences between HC and EMCI.

Our analysis was conducted on a sample of 140 patients. This number of datapoint in the context of classification using ANN and CNN is relatively small. For instance, Wang et al (2019) ^114^ used 593 samples. Having additional samples can provide more reliable results. Therefore, future research should aim to explore a larger dataset.

In this study, we proposed a method for classification of the EMCI and HC groups using graph theory. These results highlight the potential application of graph analysis of functional connectivity and efficiency of evolutionary algorithm in combination with a simple perceptron ANN in the classification of images into HC and EMCI. We proposed a fully automatic procedure for predication of early stages of AD using rs-fMRI data features. This is of particular importance considering that MRI images of EMCI individuals cannot be easily identified by experts. Further development of such methods can prove to be a powerful tool in the early diagnosis of AD.

## Supporting information

S1 Fig A sample collection of networks and regions of interests (ROI) connectivity matrix using rs-fMRI data. The colors indicate t-value for one-sample t-test statistics.
(DOCX)

S2 Fig Functional connectivity for brain areas with statistically significant correlation with other regions of interest (ROI). (DOCX)

S3 Fig All of the graph parameters on one view. (DOCX)

S4 Fig Graph parameter–average path length (the average distance from each node to any other node). (DOCX)

S5 Fig Graph parameter–betweenness centrality (the proportion of shortest paths between all node pairs in the network that pass through a given index node). (DOCX)

S6 Fig Graph parameter–clustering coefficient (the proportion of ROIs that have connectivity with a particular ROI that also have connectivity with each other). (DOCX)

S7 Fig Graph parameter–cost (the ratio of the existing number of edges to the number of all possible edges in the network). (DOCX)

S8 Fig Graph parameter–degree centrality (the number of edges that connect a node to the rest of the network). (DOCX)

S9 Fig Graph parameter–local efficiency (the network ability in transmitting information at the local level). (DOCX)

S10 Fig Graph parameter–global efficiency (the average inverse shortest path length in the network; this parameter is inversely related to the path length). (DOCX)

S11 Fig **Comparison of classification performance for 200 repetitions (light blue) and 500 repetitions (dark blue) for different optimization algorithms per parameter set.** The subplots show the difference between 200 and 500 repetitions, showing small superior performance for 500 repetitions. This is an indication that the algorithms converted within the first 200 repetitions.
(DOCX)

S1 Table Summary of the studies aiming at categorization of healthy (HC), mild cognitive impairment (MCI) and Alzheimer’s disease (AD) using different biomarkers and classification methods based on Table 1. (DOCX)

S2 Table Comparison of performance of different methods split across male and female participants. (DOCX)

S1 Methods Further description of the methods. (DOCX)

S1 File Spreadsheets containing the graph parameters, brain areas and network values. (XLSX)

S2 File Spreadsheet containing the source of data. (XLSX)
