# Supplementary material for: Classification of early-MCI patients from healthy controls using evolutionary optimization of graph measures of resting-state fMRI, for the Alzheimer’s disease neuroimaging initiative
Source: PLoS One. 2022 Jun 21;17(6):e0267608. doi: 10.1371/journal.pone.0267608 (PMC9212187; doi:10.1371/journal.pone.0267608)
Supplement: S1 Fig — The colors indicate t-value for one-sample t-test statistics. (DOCX) [file pone.0267608.s001.docx]

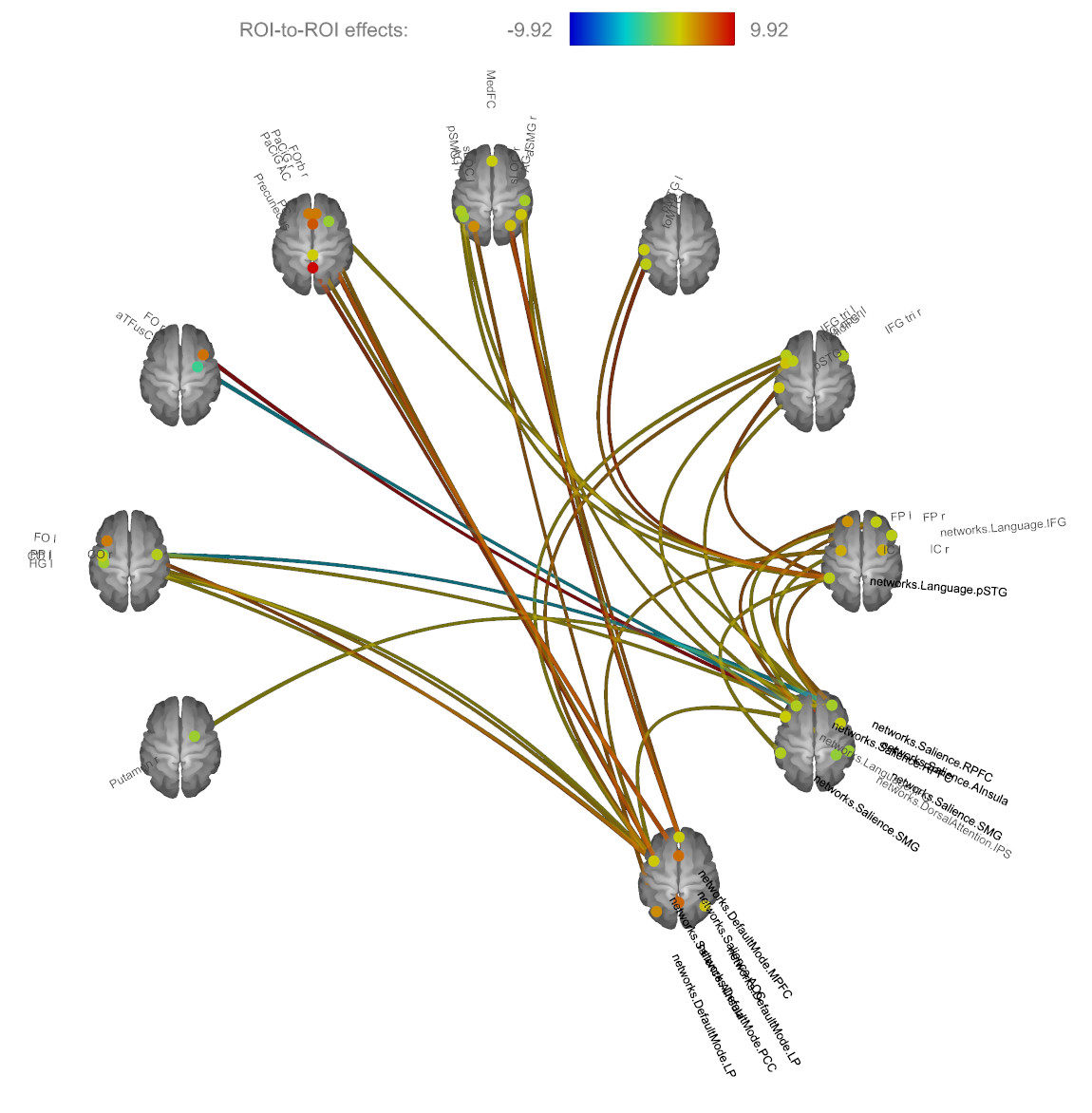


Supplementary Figure 1. A sample collection of networks and regions of interests (ROI) connectivity matrix using rs-fMRI data. The colors indicate t-value for one-sample t-test statistics.

Reference: Nieto-Castanon A. Handbook of functional connectivity magnetic resonance imaging methods in CONN. Hilbert-Press, Boston, MA. 2020.
