# Supplementary material for: Classification of early-MCI patients from healthy controls using evolutionary optimization of graph measures of resting-state fMRI, for the Alzheimer’s disease neuroimaging initiative
Source: PLoS One. 2022 Jun 21;17(6):e0267608. doi: 10.1371/journal.pone.0267608 (PMC9212187; doi:10.1371/journal.pone.0267608)
Supplement: S3 Fig — (DOCX) [file pone.0267608.s003.docx]

| 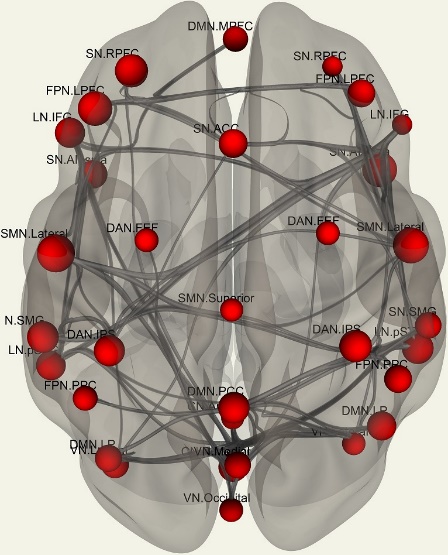 | 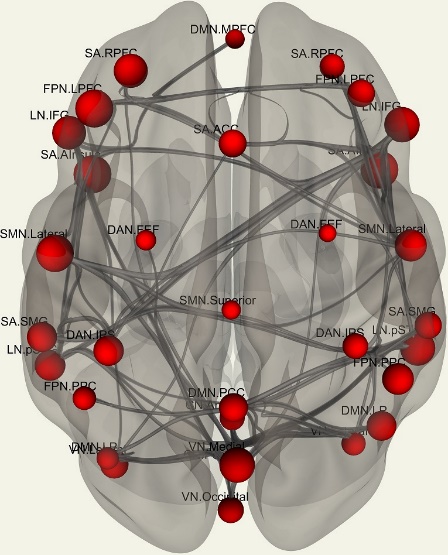 | 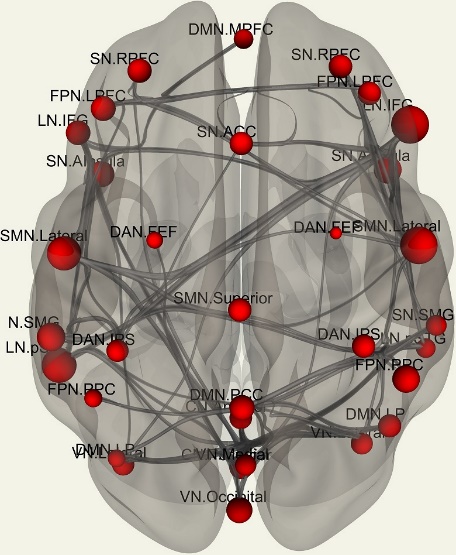 |
| --- | --- | --- |
| average path length | betweenness centrality | clustering coefficient |
| 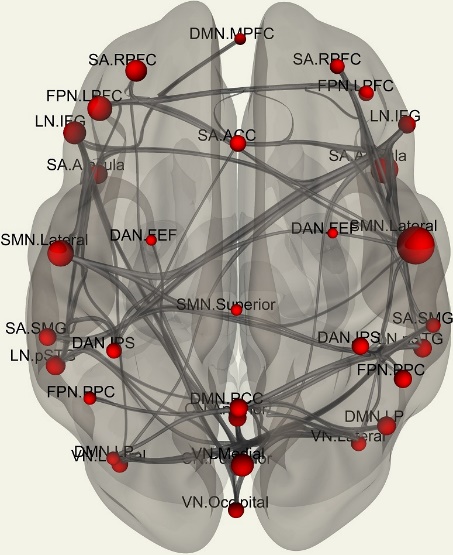 | 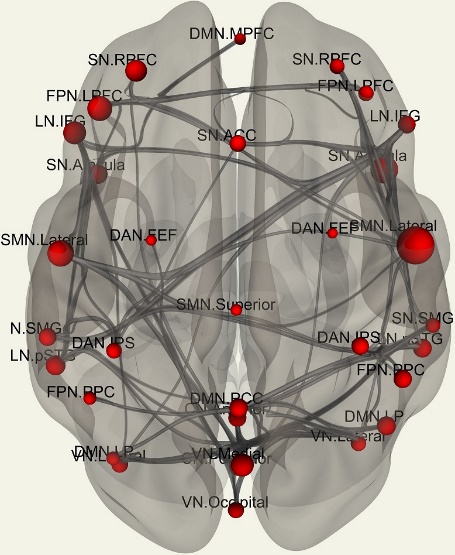 | 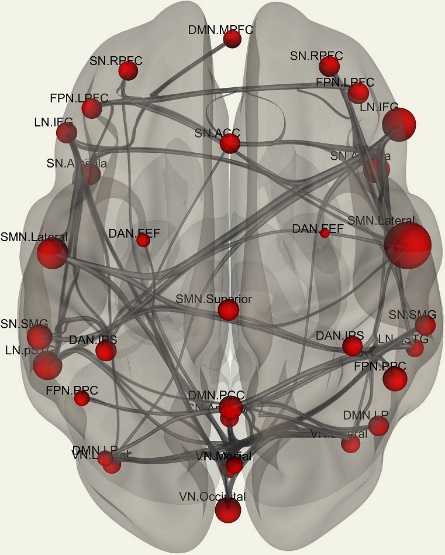 |
| cost | degree centrality | local efficiency |
| 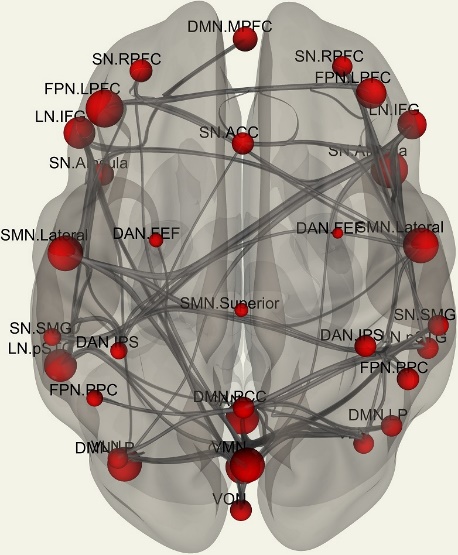 |  |  |
| global efficiency |  |  |

Supplementary Figure 3. All of the graph parameters on one view.
