# Supplementary material for: Classification of early-MCI patients from healthy controls using evolutionary optimization of graph measures of resting-state fMRI, for the Alzheimer’s disease neuroimaging initiative
Source: PLoS One. 2022 Jun 21;17(6):e0267608. doi: 10.1371/journal.pone.0267608 (PMC9212187; doi:10.1371/journal.pone.0267608)
Supplement: S4 Fig — (DOCX) [file pone.0267608.s004.docx]

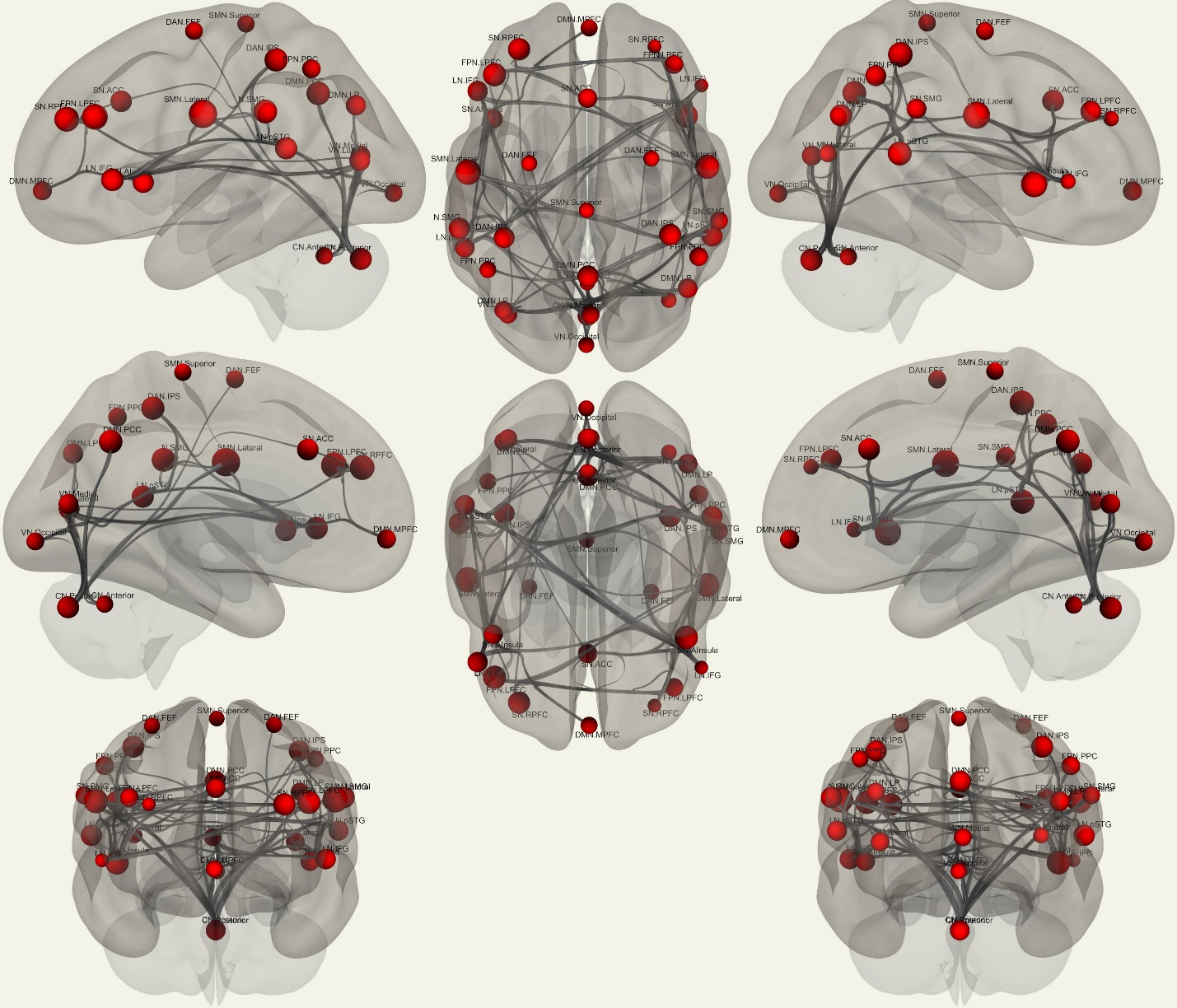


Supplementary Figure 4. Graph parameter – *average path length* (the average distance from each node to any other node)
