# Supplementary material for: Classification of early-MCI patients from healthy controls using evolutionary optimization of graph measures of resting-state fMRI, for the Alzheimer’s disease neuroimaging initiative
Source: PLoS One. 2022 Jun 21;17(6):e0267608. doi: 10.1371/journal.pone.0267608 (PMC9212187; doi:10.1371/journal.pone.0267608)
Supplement: S5 Fig — (DOCX) [file pone.0267608.s005.docx]

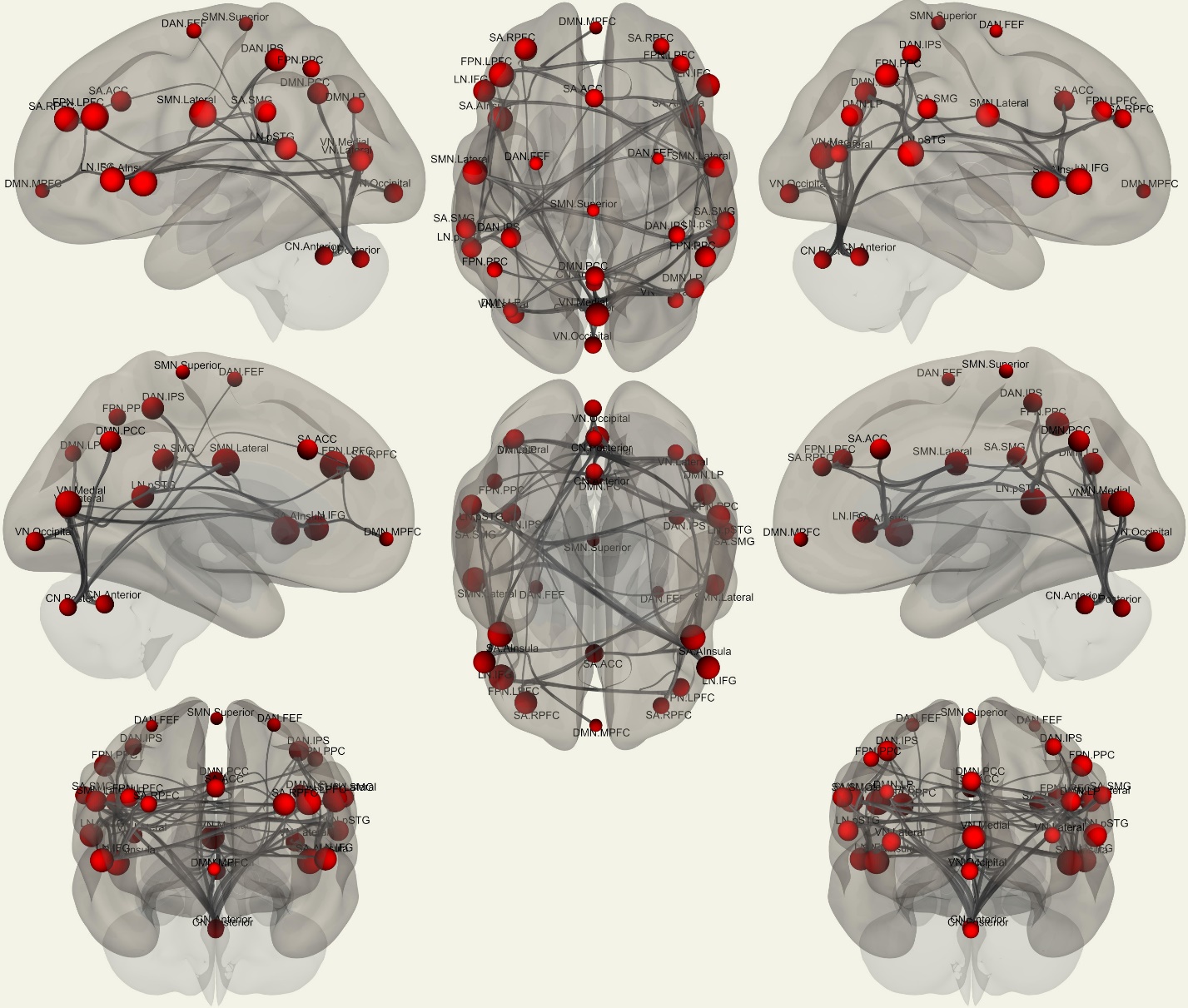


Supplementary Figure 5. Graph parameter – *betweenness centrality* (the proportion of shortest paths between all node pairs in the network that pass through a given index node)
