# Supplementary material for: Classification of early-MCI patients from healthy controls using evolutionary optimization of graph measures of resting-state fMRI, for the Alzheimer’s disease neuroimaging initiative
Source: PLoS One. 2022 Jun 21;17(6):e0267608. doi: 10.1371/journal.pone.0267608 (PMC9212187; doi:10.1371/journal.pone.0267608)
Supplement: S11 Fig — Comparison of classification performance for 200 repetitions (light blue) and 500 repetitions (dark blue) for different optimization algorithms per parameter set. The subplots show the difference between 200 and 500 repetitions, showing small superior performance for 500 repetitions. This is an indication that the algorithms converted within the first 200 repetitions. (DOCX) [file pone.0267608.s011.docx]

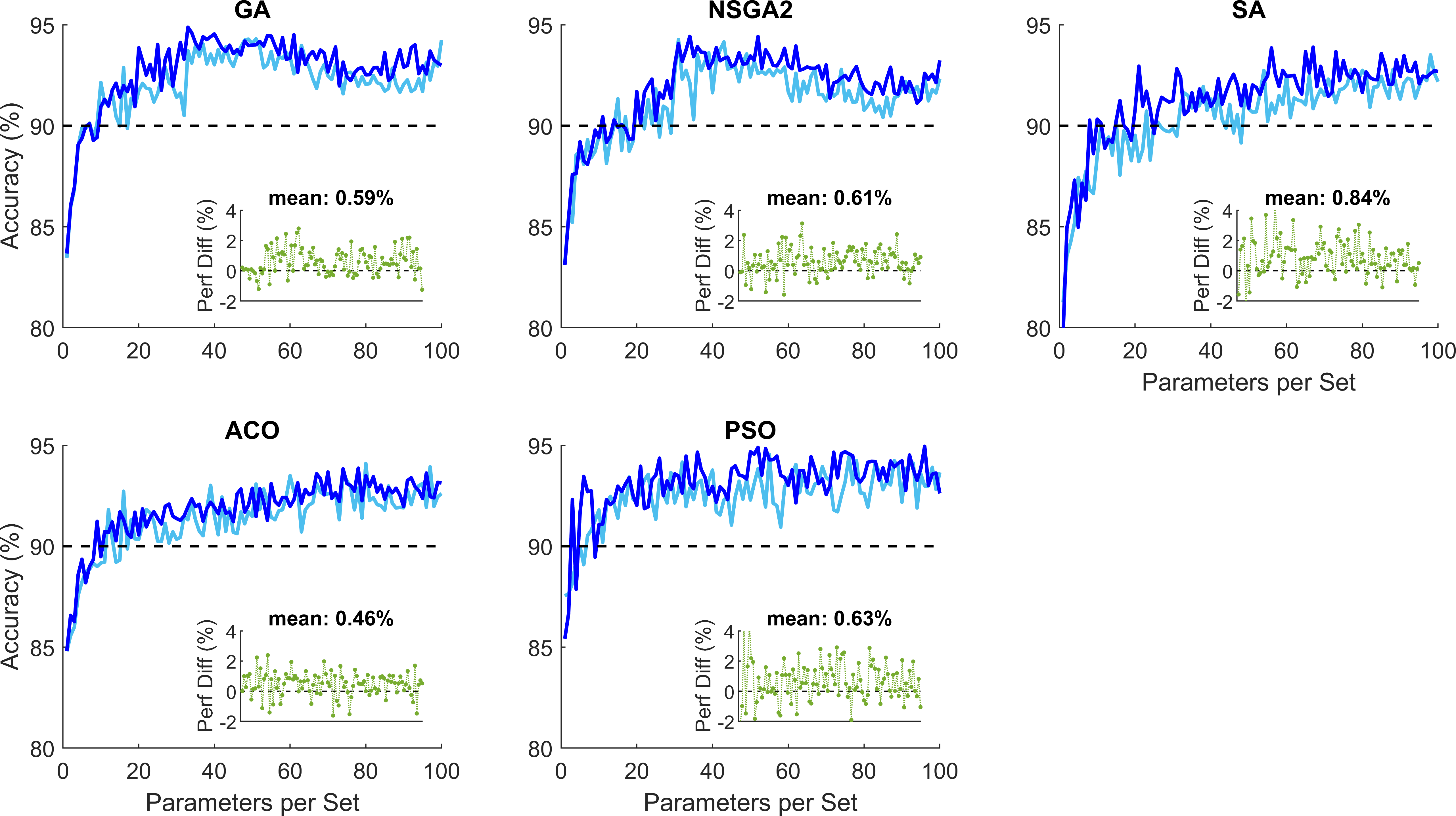


Supplementary Figure 11. Comparison of classification performance for 200 repetitions (light blue) and 500 repetitions (dark blue) for different optimization algorithms per parameter set. The subplots show the difference between 200 and 500 repetitions, showing small superior performance for 500 repetitions. This is an indication that the algorithms converted within the first 200 repetitions.
