# Supplementary material for: Classification of early-MCI patients from healthy controls using evolutionary optimization of graph measures of resting-state fMRI, for the Alzheimer’s disease neuroimaging initiative
Source: PLoS One. 2022 Jun 21;17(6):e0267608. doi: 10.1371/journal.pone.0267608 (PMC9212187; doi:10.1371/journal.pone.0267608)
Supplement: S1 Table — (DOCX) [file pone.0267608.s012.docx]

Supplementary Table 1. Summary of the studies aiming at categorization of healthy (HC), mild cognitive impairment (MCI) and Alzheimer’s disease (AD) using different biomarkers and classification methods based on Table 1.

|  |  |  |  | **HC** | **MCI** |  |  | **AD** |  |
| --- | --- | --- | --- | --- | --- | --- | --- | --- | --- |
| **Study** ↑ | **Cit.** | **Method** | **Modalities** | **n** | **Cat.** | **n** | **Acc%** | **n** | **Acc%** |
| Wolz et al (2011) | ^1^ | LDA | MRI | 231 | sMCI | 238 | 68 | 198 | 89 |
|  |  |  |  |  | cMCI | 167 | 84 |  |  |
| Zhang et al (2011) | ^2^ | SVM | MRI+ FDG-PET+ CSF | 231 | SMCI | 238 | 82 | 198 | 87 |
|  |  | LDA |  |  | PMCI | 167 | 84 |  | 89 |
| Liu et al (2012) | ^3^ | SRC | MRI | 229 | MCI | 225 | 87.85 | 198 | 90.8 |
| Gray et al (2013) | ^4^ | RF | MRI+PET+CSF+genetic | 35 | MCI | 75 | 75 | 37 | 89 |
| Liu et al (2013) | ^5^ | SVM + LLE | MRI | 137 | sMCI | 92 | 69 | 86 | 90 |
|  |  |  |  |  | cMCI | 97 | 81 |  |  |
| Wee et al (2013) | ^6^ | SVM | MRI | 200 | MCI | 200 | 83.75 | 198 | 92.35 |
| Guerrero et al (2014) | ^7^ | SVM | MRI | 134 | EMCI | 229 | 65 | 106 | 86 |
|  |  |  | MRI | 175 | cMCI | 116 | 82 | 106 |  |
| Payan & Montana (2015) | ^8^ | CNN | MRI | 755 | MCI | 755 | 92.11 | 755 | 95.39 |
| Prasad et al (2015) | ^9^ | SVM | DWI | 50 | EMCI | 74 | 59.2 | 38 | 78.2 |
|  |  |  |  |  | LMCI | 38 | 62.8 |  |  |
| Suk et al (2015) | ^10^ | DNN | MRI+PET | 52 | MCI | 99 | 90.7 | 51 | 98.8 |
| Shakeri et al (2016) | ^11^ | DNN | MRI | 150 | EMCI | 160 | 56 | 90 | 84 |
|  |  |  | MRI |  | LMCI | 160 | 59 |  |  |
| Aderghal, Benois-Pineau et al (2017) | ^12^ | CNN | MRI | 228 | MCI | 399 | 66.2 | 188 | 91.41 |
| Aderghal, Boissenin et al (2017) | ^13^ | CNN | MRI | 228 | MCI | 399 | 66 | 188 | 82.8 |
| Billones et al (2017) | ^14^ | CNN | MRI | 300 | MCI | 300 | 91.67 | 300 | 98.33 |
| Guo et al (2017) | ^15^ | SVM | rs-fMRI | 28 | EMCI | 32 | 72.8 | 38 | 88.91 |
|  |  |  |  |  | LMCI | 32 | 78.63 |  |  |
| Korolev et al (2017) | ^16^ | CNN | MRI | 61 | LMCI | 43 | 63 | 50 | 80 |
|  |  |  |  |  | EMCI | 77 | 56 |  |  |
| Wang et al (2017) | ^17^ | CNN | MRI | 229 | MCI | 400 | 90.6 |  |  |
| Li & Liu (2018) | ^18^ | CNN | MRI | 229 | MCI | 403 | 73.8 | 199 | 92.4 |
| Qiu et al (2018) | ^19^ | CNN | MRI+MMSE+LM | 303 | MCI | 83 | 90.9 |  |  |
| Senanayake et al (2018) | ^20^ | CNN | MRI+ NM | 161 | MCI | 193 | 75 | 161 | 79 |
| Altaf et al (2018) | ^21^ | SVM | MRI | 90 | MCI | 105 | 79.8 | 92 | 58 |
|  |  | Ensemble | MRI |  | MCI |  | 75 |  | 58 |
|  |  | KNN | MRI |  | MCI |  | 75 |  | 56 |
|  |  | Tree | MRI |  | MCI |  | 78 |  | 59 |
|  |  | SVM | clinical+MRI |  | MCI |  | 83 |  | 98 |
|  |  | Ensemble | clinical+MRI |  | MCI |  | 82 |  | 98 |
|  |  | KNN | clinical+MRI |  | MCI |  | 86 |  | 92 |
|  |  | Tree | clinical+MRI |  | MCI |  | 80 |  | 98 |
| Forouzannezhad et al (2018) | ^22^ | SVM | MRI | 248 | EMCI | 296 | 73.1 | 159 | 90.3 |
|  |  |  | MRI |  | LMCI | 193 | 63 |  |  |
|  |  |  | PET |  | LMCI |  | 73.6 |  | 82.5 |
|  |  |  | PET+MRI |  | LMCI |  | 76.9 |  | 91.2 |
|  |  |  | PET+MRI |  | EMCI |  | 75.6 |  |  |
|  |  |  | PET+MRI+NTS |  | LMCI |  | 91.9 |  | 96.2 |
|  |  |  | PET+MRI+NTS |  | EMCI |  | 81.1 |  |  |
| Hosseini Asl et al (2018) | ^23^ | CNN | MRI | 70 | MCI | 70 | 94 | 70 | 99 |
| Jie, Liu, Shen et al (2018) | ^24^ | SVM | rs-fMRI | 50 | EMCI | 56 | 78.3 |  |  |
| Jie, Liu, Zhang et al (2018) | ^25^ | SVM | rs-fMRI | 50 | MCI | 99 | 82.6 |  |  |
| Raeper et al (2018) | ^26^ | SVM + LDA | MRI | 42 | EMCI | 42 | 80.95 |  |  |
| Basaia et al (2019) | ^27^ | CNN | MRI | 407 | cMCI | 280 | 87.1 | 418 | 99 |
|  |  |  |  |  | sMCI | 533 | 76.1 |  |  |
| Forouzannezhad et al (2019) | ^28^ | DNN | MRI | 248 | EMCI | 296 | 61.1 | 159 | 82.2 |
|  |  |  | MRI |  | LMCI | 193 | 64.1 |  |  |
|  |  |  | PET |  | EMCI |  | 58.2 |  | 88.9 |
|  |  |  | PET |  | LMCI |  | 66 |  |  |
|  |  |  | MRI+PET |  | EMCI |  | 68 |  | 89.6 |
|  |  |  | MRI+PET |  | LMCI |  | 71.7 |  |  |
|  |  |  | MRI+PET+NTS |  | EMCI |  | 84 |  | 96.8 |
|  |  |  | MRI+PET+NTS |  | LMCI |  | 84.1 |  |  |
| Wang et al (2019) | ^29^ | DNN | MRI | 209 | MCI | 384 | 98.42 | 240 | 98.83 |
| Wee et al (2019) | ^30^ | CNN | MRI | 300 | LMCI | 208 | 69.3 | 261 | 85.8 |
|  |  |  |  |  | EMCI | 314 | 51.8 |  |  |
|  |  |  |  | 242 | MCI | 415 | 67.6 | 355 | 81 |
| Kam et al (2020) | ^31^ | CNN | rs-fMRI | 48 | EMCI | 49 | 76.07 |  |  |
| Fang et al (2020) | ^32^ | GDCA | MRI+PET | 251 | EMCI |  | 79.25 |  |  |
| Forouzannezhad et al (2020) | ^33^ | GP | MRI | 248 | EMCI | 296 | 75.9 | 159 | 83.6 |
|  |  |  | MRI |  | LMCI | 193 | 62.1 |  |  |
|  |  |  | MRI+PET |  | EMCI |  | 75.9 |  | 92.5 |
|  |  |  | MRI+PET |  | LMCI |  | 78.1 |  |  |
|  |  |  | MRI+PET+DI |  | EMCI |  | 78.8 |  | 94.7 |
|  |  |  | MRI+PET+DI |  | LMCI |  | 79.8 |  |  |
|  |  |  | PET |  | LMCI |  | 76.1 |  | 90 |
| Jiang et al (2020) | ^34^ | CNN | MRI | 50 | EMCI | 70 | 89.4 |  |  |
| Kang et al (2020) | ^35^ | CNN | DTI | 50 | EMCI | 70 | 71.7 |  |  |
|  |  | CNN | MRI |  | EMCI |  | 73.3 |  |  |
|  |  |  | DTI+MRI |  | EMCI |  | 94.2 |  |  |
| Yang et al (2021) | ^36^ | SVM | rs-fMRI | 29 | EMCI | 29 | 82.76 |  |  |
|  |  |  |  |  | LMCI | 18 | 87.23 |  |  |
| **our method** |  | **EA + ANN** | **rs-fMRI** | **68** | **EMCI** | **72** | **94.5** |  |  |

Notes: ↑ table sorted based on the year of publication. Acc: classification accuracy percentage between MCI and HC groups; ANN: artificial neural networks; Cat.: category of MCI; Cit.: citation; cMCI: MCI converted to AD; CNN: convolutional neural networks; DI: demographic information; DNN: deep neural network; DTI: diffusion tensor imaging; DWI: diffusion-weighted imaging; EA: evolutionary algorithms; EMCI: early-MCI; GDCA: gaussian discriminative component analysis; GP: gaussian process; KNN: K nearest neighbors; LDA: linear discriminative analysis; LLE: locally linear embedding; LM: logical memory; LMCI: late-MCI; MMSE: mini-mental state examination; NM: neuropsychological measures; NTS: neuropsychological test scores; PET: positron emission therapy; rs-fMRI: resting-state fMRI; sMCI: stable MCI; SRC: sparse representation-based classifier; SVM: support vector machine.

Supplementary Table 2. Comparison of performance of different methods split across male and female participants.

| Methods | Male (%) | Female (%) | Overall (%) |
| --- | --- | --- | --- |
| GA | 95.00 | 94.00 | 94.50 |
| NSGA2 | 94.00 | 95.00 | 94.50 |
| SA | 94.00 | 94.50 | 94.25 |
| ACO | 92.50 | 96.00 | 94.25 |
| PSO | 93.00 | 96.50 | 94.75 |
| Statistical | 86.00 | 90.00 | 88.00 |

# References

1.         Wolz, R. *et al.* Multi-method analysis of MRI images in early diagnostics of Alzheimer’s disease. *PLoS ONE* **6**, 1–9 (2011).

2.         Zhang, D., Wang, Y., Zhou, L., Yuan, H. & Shen, D. Multimodal classification of Alzheimer’s disease and mild cognitive impairment. *NeuroImage* **55**, 856–867 (2011).

3.         Liu, M., Zhang, D. & Shen, D. Ensemble sparse classification of Alzheimer’s disease. *NeuroImage* **60**, 1106–1116 (2012).

4.         Gray, K. R., Aljabar, P., Heckemann, R. A., Hammers, A. & Rueckert, D. Random forest-based similarity measures for multi-modal classification of Alzheimer’s disease. *NeuroImage* **65**, 167–175 (2013).

5.         Liu, X., Tosun, D., Weiner, M. W. & Schuff, N. Locally linear embedding (LLE) for MRI based Alzheimer’s disease classification. *NeuroImage* **83**, 148–157 (2013).

6.         Wee, C. Y., Yap, P. T. & Shen, D. Prediction of Alzheimer’s disease and mild cognitive impairment using cortical morphological patterns. *Human Brain Mapping* **34**, 3411–3425 (2013).

7.         Guerrero, R., Wolz, R., Rao, A. W. & Rueckert, D. Manifold population modeling as a neuro-imaging biomarker: Application to ADNI and ADNI-GO. *NeuroImage* **94**, 275–286 (2014).

8.         Payan, A. & Montana, G. Predicting Alzheimer’s disease a neuroimaging study with 3D convolutional neural networks. *ICPRAM 2015 - 4th International Conference on Pattern Recognition Applications and Methods, Proceedings* **2**, 355–362 (2015).

9.         Prasad, G., Joshi, S. H., Nir, T. M., Toga, A. W. & Thompson, P. M. Brain connectivity and novel network measures for Alzheimer’s disease classification. *Neurobiology of Aging* **36**, S121–S131 (2015).

10.        Suk, H. il, Lee, S. W. & Shen, D. Latent feature representation with stacked auto-encoder for AD/MCI diagnosis. *Brain Structure and Function* **220**, 841–859 (2015).

11.        Shakeri, M., Lombaert, H., Tripathi, S. & Kadoury, S. Deep spectral-based shape features for Alzheimer’s disease classification. *Lecture Notes in Computer Science (including subseries Lecture Notes in Artificial Intelligence and Lecture Notes in Bioinformatics)* **10126 LNCS**, 15–24 (2016).

12.        Aderghal, K., Benois-Pineau, J., Afdel, K. & Gwenaëlle, C. FuseMe: Classification of sMRI images by fusion of deep CNNs in 2D+e projections. *ACM International Conference Proceeding Series* **Part F1301**, (2017).

13.        Aderghal, K., Boissenin, M., Benois-Pineau, J., Catheline, G. & Afdel, K. Classification of sMRI for AD Diagnosis with Convolutional Neuronal Networks: A Pilot 2-D+ ϵ Study on ADNI. in *MultiMedia Modeling, Lecture Notes in Computer Science* (eds. Amsaleg, L., Gu ðmundsson, G., Gurrin, C., Jónsson, B. & Satoh, S.) 690–701 (Springer International Publishing, 2017). doi:10.1007/978-3-319-51811-4_56.

14.        Billones, C. D., Demetria, O. J. L. D., Hostallero, D. E. D. & Naval, P. C. DemNet: A Convolutional Neural Network for the detection of Alzheimer’s Disease and Mild Cognitive Impairment. *IEEE Region 10 Annual International Conference, Proceedings/TENCON* 3724–3727 (2017) doi:10.1109/TENCON.2016.7848755.

15.        Guo, H., Zhang, F., Chen, J., Xu, Y. & Xiang, J. Machine learning classification combining multiple features of a hyper-network of fMRI data in Alzheimer’s disease. *Frontiers in Neuroscience* **11**, 1–22 (2017).

16.        Korolev, S., Safiullin, A., Belyaev, M. & Dodonova, Y. Residual and plain convolutional neural networks for 3D brain MRI classification. *arXiv* 835–838 (2017).

17.        Wang, S., Shen, Y., Chen, W., Xiao, T. & Hu, J. Automatic Recognition of Mild Cognitive Impairment from MRI Images Using Expedited Convolutional Neural Networks. in *Lecture Notes in Computer Science (including subseries Lecture Notes in Artificial Intelligence and Lecture Notes in Bioinformatics)* vol. 10613 LNCS 373–380 (2017).

18.        Li, F. & Liu, M. Alzheimer’s disease diagnosis based on multiple cluster dense convolutional networks. *Computerized Medical Imaging and Graphics* **70**, 101–110 (2018).

19.        Qiu, S. *et al.* Fusion of deep learning models of MRI scans, Mini–Mental State Examination, and logical memory test enhances diagnosis of mild cognitive impairment. *Alzheimer’s and Dementia: Diagnosis, Assessment and Disease Monitoring* **10**, 737–749 (2018).

20.        Senanayake, U., Sowmya, A. & Dawes, L. Deep fusion pipeline for mild cognitive impairment diagnosis. in *2018 IEEE 15th International Symposium on Biomedical Imaging (ISBI 2018)* 1394–1997 (IEEE, 2018). doi:10.1109/ISBI.2018.8363832.

21.        Altaf, T., Anwar, S. M., Gul, N., Majeed, M. N. & Majid, M. Multi-class Alzheimer’s disease classification using image and clinical features. *Biomedical Signal Processing and Control* **43**, 64–74 (2018).

22.        Forouzannezhad, P., Abbaspour, A., Cabrerizo, M. & Adjouadi, M. Early Diagnosis of Mild Cognitive Impairment Using Random Forest Feature Selection. in *2018 IEEE Biomedical Circuits and Systems Conference (BioCAS)* vol. 53 1–4 (IEEE, 2018).

23.        Hosseini Asl, E. *et al.* Alzheimer rsquo s disease diagnostics by a 3D deeply supervised adaptable convolutional network. *Frontiers in Bioscience* **23**, 584–596 (2018).

24.        Jie, B., Liu, M. & Shen, D. Integration of temporal and spatial properties of dynamic connectivity networks for automatic diagnosis of brain disease. *Medical Image Analysis* **47**, 81–94 (2018).

25.        Jie, B., Liu, M., Zhang, D. & Shen, D. Sub-Network Kernels for Measuring Similarity of Brain Connectivity Networks in Disease Diagnosis. *IEEE Transactions on Image Processing* **27**, 2340–2353 (2018).

26.        Raeper, R., Lisowska, A. & Rekik, I. Cooperative Correlational and Discriminative Ensemble Classifier Learning for Early Dementia Diagnosis Using Morphological Brain Multiplexes. *IEEE Access* **6**, 43830–43839 (2018).

27.        Basaia, S. *et al.* Automated classification of Alzheimer’s disease and mild cognitive impairment using a single MRI and deep neural networks. *NeuroImage: Clinical* **21**, 101645 (2019).

28.        Forouzannezhad, P., Abbaspour, A., Li, C., Cabrerizo, M. & Adjouadi, M. A Deep Neural Network Approach for Early Diagnosis of Mild Cognitive Impairment Using Multiple Features. *Proceedings - 17th IEEE International Conference on Machine Learning and Applications, ICMLA 2018* 1341–1346 (2019) doi:10.1109/ICMLA.2018.00218.

29.        Wang, H. *et al.* Ensemble of 3D densely connected convolutional network for diagnosis of mild cognitive impairment and Alzheimer’s disease. *Neurocomputing* **333**, 145–156 (2019).

30.        Wee, C. Y. *et al.* Cortical graph neural network for AD and MCI diagnosis and transfer learning across populations. *NeuroImage: Clinical* **23**, 101929 (2019).

31.        Kam, T. E., Zhang, H., Jiao, Z. & Shen, Di. Deep Learning of Static and Dynamic Brain Functional Networks for Early MCI Detection. *IEEE Transactions on Medical Imaging* **39**, 478–487 (2020).

32.        Fang, C. *et al.* Gaussian discriminative component analysis for early detection of Alzheimer’s disease: A supervised dimensionality reduction algorithm. *Journal of Neuroscience Methods* **344**, 108856 (2020).

33.        Forouzannezhad, P. *et al.* A Gaussian-based model for early detection of mild cognitive impairment using multimodal neuroimaging. *Journal of Neuroscience Methods* **333**, 108544 (2020).

34.        Jiang, J., Kang, L., Huang, J. & Zhang, T. Deep learning based mild cognitive impairment diagnosis using structure MR images. *Neuroscience Letters* **730**, 134971 (2020).

35.        Kang, L., Jiang, J., Huang, J. & Zhang, T. Identifying Early Mild Cognitive Impairment by Multi-Modality MRI-Based Deep Learning. *Frontiers in Aging Neuroscience* **12**, 1–10 (2020).

36.        Yang, P. *et al.* Fused Sparse Network Learning for Longitudinal Analysis of Mild Cognitive Impairment. *IEEE Transactions on Cybernetics* **51**, 233–246 (2021).
