# Supplementary material for: Classification of early-MCI patients from healthy controls using evolutionary optimization of graph measures of resting-state fMRI, for the Alzheimer’s disease neuroimaging initiative
Source: PLoS One. 2022 Jun 21;17(6):e0267608. doi: 10.1371/journal.pone.0267608 (PMC9212187; doi:10.1371/journal.pone.0267608)
Supplement: S2 Table — (DOCX) [file pone.0267608.s013.docx]

Supplementary Table 2. Comparison of performance of different methods split across male and female participants.

| Methods | Male (%) | Female (%) | Overall (%) |
| --- | --- | --- | --- |
| GA | 95.00 | 94.00 | 94.50 |
| NSGA2 | 94.00 | 95.00 | 94.50 |
| SA | 94.00 | 94.50 | 94.25 |
| ACO | 92.50 | 96.00 | 94.25 |
| PSO | 93.00 | 96.50 | 94.75 |
| Statistical | 86.00 | 90.00 | 88.00 |
