# Supplementary material for: Classification of early-MCI patients from healthy controls using evolutionary optimization of graph measures of resting-state fMRI, for the Alzheimer’s disease neuroimaging initiative
Source: PLoS One. 2022 Jun 21;17(6):e0267608. doi: 10.1371/journal.pone.0267608 (PMC9212187; doi:10.1371/journal.pone.0267608)
Supplement: S1 Methods — (DOCX) [file pone.0267608.s014.docx]

Supplementary Methods

# Genetic algorithm (GA)

The procedure of GA consists of the following four steps ^1^:

1. *Individual encoding*: Each individual is encoded as binary vector of size $P$, where the entry $b_{i}=1$ states for the predictor $p_{i}$ that is defined for that individual, $b_{i}=0$ if the predictor $p_{i}$ is not included in that particular individual ($i=1,\ldots,P$).
2. *Initial population*: Given the binary representation of the individuals, the population is a binary matrix where its rows are the randomly selected individuals, and the columns are the available predictors. An initial population with a predefined number of individuals is generated with a random selection of 0 and 1 for each entry.
3. *Fitness function*: the fitness value of the individual in the population is calculated using predefined fitness function. Individual with the lowest prediction error and fewer predictors have been selected for next generation.
4. *Genetic operators*: applying genetic operators to create the next generation.

The genetic operators are, *Selection* (randomly selection of members based on their fitness value; fitter members are more likely to be chosen), *Crossover* (the new generation is created by exchanging elements between two selected parents from the previous step), *Mutation* (elements in a selected member is changed), and *Stop Criteria* (the criteria and indicate the end of the search) ^1^. In our study we used roulette wheel selection for selection of the possible valuable solutions to producing offsprings for the next generation.

# Nondominated sorting genetic algorithm II (NSGA-II)

Nondomination and crowding distance are used to sort the new members. A specific number of individuals in the sorted population are transferred to the next generation. This conventional NSGA algorithm has a computational complexity of $O({MN}^{3})$, where $M$ is the number of objectives and $N$ is the population size. NSGA-II on the other hand has overall complexity $O\left( {MN}^{2} \right)$, which is significantly ^2^. After termination of the optimization process, nondominated solutions form the Pareto frontier. Each of the solutions on the Pareto frontier can be considered as an optimal strategy for a specific situation ^3–5^.

# Ant colony optimization algorithm (ACO)

See Figure 1 for the procedure of the traverse of an ant placed at node $a$. This ant has a choice of which feature to add next to its path (dotted lines). It traverses through the graph to find a path that satisfies the stopping criterion (e.g., a suitably high classification accuracy has been achieved with this subset). In this example, the ant chooses next feature $b$ based on a set of transition rules, then $c$ and then $d$. Upon arrival at$d$, the current subset $\{a;b;c;d\}$ is determined to satisfy the traversal stopping criterion. At termination of search, the algorithm outputs this feature subset as a candidate for data reduction ^9^.


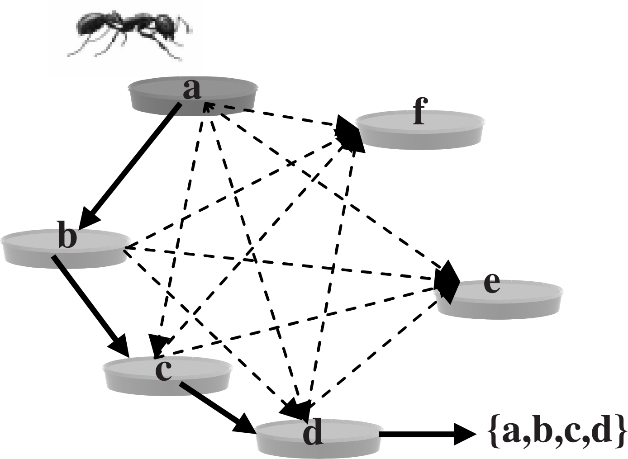


Figure 1. A sample example of ant traveling through multiple features in ant colony optimization algorithm (ACO). Here feature subset of $\{a;b;c;d\}$ is selected as a possible solution ^9^.

The probability of an ant at feature $i$ choosing to travel to feature $j$ at time $t$:

$$p_{ij}^{n}\left( t \right)=\left\{ \begin{matrix} \frac{\left[ \vartheta_{ij}\left( t \right) \right]^{\alpha}.\left[ \varphi_{ij} \right]^{\beta}}{\sum_{l\in J_{i}^{n}} \left[ \vartheta_{ij}\left( t \right) \right]^{\alpha}.\left[ \varphi_{ij} \right]^{\beta}} & \text{if} j\in J_{i}^{n} \\ 0 & \text{otherwise} \end{matrix} \right.$$

where $n$ is the number of ants, $\varphi_{ij}$ is the heuristic desirability of choosing feature $j$ when at feature $i$, $J_{i}^{n}$ is the set of nodes next to node $i$, which have not yet been visited by the ant $n$. The $\alpha>0$ and $\beta>0$ are two parameters that determine the relative importance of the pheromone value and heuristic information, respectively, and $\vartheta_{ij}$is the amount of virtual pheromone on edge $(i,j)$. The pheromone on each edge is updated according to the following formula ^9^:

$$\vartheta_{ij}\left( t+1 \right)=\left( 1-\rho\right)\vartheta_{ij}\left( t \right)+\rho\Delta\vartheta_{ij}\left( t \right)$$

$$\Delta\vartheta_{ij}\left( t \right)=\sum_{n=1}^{N} (\gamma(F^{n})/\left| F^{n} \right|)$$

This is the case if the edge $(i,j)$ has been traversed; $\Delta\vartheta_{ij}\left( t \right)$ is 0 otherwise. The value $0\leq\rho\leq1$ is decay constant used to simulate the evaporation of the pheromone. The pheromone is updated according to both the measure of the "goodness" of the ant's feature subset $\gamma$ and the size of the subset itself. By this definition, all ants update the pheromone ^9^. $F^{n}$ is the feature subset found by ant $n$.

# Simulated annealing (SA)

SA utilizes a certain probability to accept a worse solution. The algorithm starts with a randomly generated solution; in each iteration, a neighbor solution to the best solution so far is generated according to a predefined neighborhood structure and evaluated using a fitness function. The improving move is accepted, whilst worse neighbors are accepted with a certain probability determined by the Boltzmann probability, $P=e-\theta/ T$ where $\theta$ is the difference between the fitness of the best solution and the generated neighbor. Moreover, $T$ is a temperature, which periodically decreases during the search process according to some cooling schedule. First, the current temperature $T$ is set to be a very large number ^13,14^.

# Particle swarm optimization (PSO)

In a PSO with an N-dimensional search space, the particle position and velocity are formulated by:

$$V_{ij}=\left| w\times x_{ij}+c_{p}\times r_{p}\times\left( p_{ij}-x_{ij} \right)+c_{g}\times r_{g}\times\left( p_{gj}-x_{ij} \right) \right|$$

$$x_{ij}=x_{ij}+v_{ij},$$

where $V_{i}$ and $x_{i}$ refer to the velocity and position of the particle $i$, respectively, and $j$, ranging from 1 to N (total number of features). $c_{p}$ is the cognitive parameter, defining the degree of acceleration towards the particle’s individual local best position $p_{ij}$. $c_{g}$ is a social parameter, defining the acceleration towards the global best position $p_{gj}$. $w$ is an inertia parameter, regulating the overall rate of change. The stochastic nature of the velocity equation is represented by $r_{p}$ and$r_{g}$, which are numbers in the range [0, 1]. To maintain coherence in the swarm, the maximum velocity is regulated by a parameter $v_{max}$. In standard PSO implementations, typically $v_{max}=\left| x_{max}-x_{min} \right|$.

# References

1.         Amini, F. & Hu, G. A two-layer feature selection method using Genetic Algorithm and Elastic Net. *Expert Systems with Applications* **166**, 114072 (2021).

2.         Deb, K., Pratap, A., Agarwal, S. & Meyarivan, T. A fast and elitist multiobjective genetic algorithm: NSGA-II. *IEEE Transactions on Evolutionary Computation* **6**, 182–197 (2002).

3.         Srinivas, N. & Deb, K. Muiltiobjective Optimization Using Nondominated Sorting in Genetic Algorithms. *Evolutionary Computation* **2**, 221–248 (1994).

4.         Heris, S. M. K. & Khaloozadeh, H. Open-and closed-loop multiobjective optimal strategies for HIV therapy using NSGA-II. *IEEE Transactions on Biomedical Engineering* **58**, 1678–1685 (2011).

5.         Dang, V. Q. & Lam, C. NSC-NSGA2: Optimal search for finding multiple thresholds for nearest shrunken centroid. in *2013 IEEE International Conference on Bioinformatics and Biomedicine* 367–372 (IEEE, 2013). doi:10.1109/BIBM.2013.6732520.

6.         Dorigo, M., Caro, G. di & Gambardella, L. M. Ant Algorithms for Discrete Optimization. *Artificial Life* **5**, 137–172 (1999).

7.         Akhtar, A. Evolution of Ant Colony Optimization Algorithm — A Brief Literature Review. *arXiv* (2019).

8.         Kalami Heris, S. M. & Khaloozadeh, H. Ant Colony Estimator: An intelligent particle filter based on ACO ℝ. *Engineering Applications of Artificial Intelligence* **28**, 78–85 (2014).

9.         Kanan, H. R., Faez, K. & Taheri, S. M. Feature Selection Using Ant Colony Optimization (ACO): A New Method and Comparative Study in the Application of Face Recognition System. in *Advances in Data Mining. Theoretical Aspects and Applications* vol. 4597 LNCS 63–76 (Springer Berlin Heidelberg, 2007).

10.        Kirkpatrick, S., Gelatt, C. D. & Vecchi, M. P. Optimization by Simulated Annealing. *Science* **220**, 671–680 (1983).

11.        Anily, S. & Federgruen, A. Simulated Annealing Methods With General Acceptance Probabilities. *Journal of Applied Probability* **24**, 657–667 (1987).

12.        Bertsimas, D. & Tsitsiklis, J. Simulated annealing. *Statistical Science* **8**, 10–15 (1993).

13.        Lin, S. W., Lee, Z. J., Chen, S. C. & Tseng, T. Y. Parameter determination of support vector machine and feature selection using simulated annealing approach. *Applied Soft Computing Journal* **8**, 1505–1512 (2008).

14.        Mafarja, M. M. & Mirjalili, S. Hybrid Whale Optimization Algorithm with simulated annealing for feature selection. *Neurocomputing* **260**, 302–312 (2017).

15.        Kennedy, J. & Eberhart, R. Particle swarm optimization. in *Proceedings of ICNN’95 - International Conference on Neural Networks* vol. 4 1942–1948 (IEEE, 1995).

16.        Wang, X., Yang, J., Teng, X., Xia, W. & Jensen, R. Feature selection based on rough sets and particle swarm optimization. *Pattern Recognition Letters* **28**, 459–471 (2007).

17.        Team, Y. Particle swarm optimization in MATLAB. (2015).
